# Strong Seasonality in Arctic Estuarine Microbial Food Webs

**DOI:** 10.3389/fmicb.2019.02628

**Published:** 2019-11-29

**Authors:** Colleen T. E. Kellogg, James W. McClelland, Kenneth H. Dunton, Byron C. Crump

**Affiliations:** ^1^Hakai Institute, Heriot Bay, BC, Canada; ^2^Marine Science Institute, University of Texas at Austin, Port Aransas, TX, United States; ^3^College of Earth, Ocean, and Atmospheric Sciences, Oregon State University, Corvallis, OR, United States

**Keywords:** Arctic, Beaufort Sea, bacteria, seasonal dynamics, coastal lagoons, co-occurrence network, 16S rRNA gene, 18S rRNA gene

## Abstract

Microbial communities in the coastal Arctic Ocean experience extreme variability in organic matter and inorganic nutrients driven by seasonal shifts in sea ice extent and freshwater inputs. Lagoons border more than half of the Beaufort Sea coast and provide important habitats for migratory fish and seabirds; yet, little is known about the planktonic food webs supporting these higher trophic levels. To investigate seasonal changes in bacterial and protistan planktonic communities, amplicon sequences of 16S and 18S rRNA genes were generated from samples collected during periods of ice-cover (April), ice break-up (June), and open water (August) from shallow lagoons along the eastern Alaska Beaufort Sea coast from 2011 through 2013. Protist communities shifted from heterotrophic to photosynthetic taxa (mainly diatoms) during the winter–spring transition, and then back to a heterotroph-dominated summer community that included dinoflagellates and mixotrophic picophytoplankton such as *Micromonas* and *Bathycoccus*. Planktonic parasites belonging to Syndiniales were abundant under ice in winter at a time when allochthonous carbon inputs were low. Bacterial communities shifted from coastal marine taxa (Oceanospirillaceae, Alteromonadales) to estuarine taxa (*Polaromonas*, Bacteroidetes) during the winter-spring transition, and then to oligotrophic marine taxa (SAR86, SAR92) in summer. Chemolithoautotrophic taxa were abundant under ice, including iron-oxidizing Zetaproteobacteria. These results suggest that wintertime Arctic bacterial communities capitalize on the unique biogeochemical gradients that develop below ice near shore, potentially using chemoautotrophic metabolisms at a time when carbon inputs to the system are low. Co-occurrence networks constructed for each season showed that under-ice networks were dominated by relationships between parasitic protists and other microbial taxa, while spring networks were by far the largest and dominated by bacteria-bacteria co-occurrences. Summer networks were the smallest and least connected, suggesting a more detritus-based food web less reliant on interactions among microbial taxa. Eukaryotic and bacterial community compositions were significantly related to trends in concentrations of stable isotopes of particulate organic carbon and nitrogen, among other physiochemical variables such as dissolved oxygen, salinity, and temperature. This suggests the importance of sea ice cover and terrestrial carbon subsidies in contributing to seasonal trends in microbial communities in the coastal Beaufort Sea.

## Introduction

Aquatic microorganisms drive global cycling of carbon, nitrogen, and many other elements by carrying out key ecosystem functions including primary production, organic matter remineralization, and transformations of inorganic compounds (Falkowski et al., 2008; Ferrera et al., 2015; Worden et al., 2015). The efficiency with which microbes perform these functions is undoubtedly influenced by their physical and chemical environment (Gilbert et al., 2012), but also by interactions with each other within microbial communities (Azam and Malfatti, 2007; Fuhrman et al., 2015; Guidi et al., 2016). The composition and function of microbial communities varies strongly with seasonal changes in coastal ecosystems including day length, solar radiation, temperature, and salinity (Gilbert et al., 2012; Cram et al., 2015; Bunse and Pinhassi, 2017), and in polar regions these seasonal changes are particularly extreme, with additional complexities including ice cover and wide variations in river runoff (Holmes et al., 2012). Climate change is warming the Arctic approximately two times faster than lower latitudes (Serreze and Barry, 2011), and is amplifying seasonal variations in temperature (Serreze and Barry, 2011), ice extent (Stroeve et al., 2012), and river flow (McClelland et al., 2006; Morison et al., 2012). Moreover, increased river runoff in spring is accelerating coastal ice melt (Whitefield et al., 2015), particularly along the extensive Arctic continental shelf, where the interplay between these variables influences the timing and magnitude of biological production (Arrigo and van Dijken, 2015; Marchese et al., 2017), the species composition of primary producers (Li et al., 2009; Ardyna et al., 2014), and, in turn, higher and lower trophic levels (Wassmann et al., 2011; Vernet et al., 2017). Establishing the baseline relationship between microbial communities in Arctic coastal waters and their physical and chemical environment is key to understanding and predicting how they will respond to continued climate-induced changes to the Arctic system.

Most investigations of seasonality in microbial community composition and function in the Arctic Ocean have focused on offshore regions in the Chukchi and Canadian Beaufort Seas, the Norwegian Coast, and the plumes of very large Arctic rivers (Alonso-Sáez et al., 2008; Garneau et al., 2008; Ghiglione et al., 2012; Marquardt et al., 2016; Onda et al., 2017). Less is known about shallow estuarine environments on Arctic coastlines, despite their importance to coastal fisheries (von Biela et al., 2013) and as breeding habitat for over 157 species of migrating birds (Brown et al., 2007). Nearly one-half the Alaskan Beaufort Sea coast and one-third of the Chukchi Sea coast is skirted by an irregular and discontinuous chain of barrier islands that enclose shallow (< 6 m deep) lagoons (Dunton et al., 2006; Schreiner et al., 2013). Seasonal changes in these lagoons are different than in the open Arctic Ocean. For example, the magnitude of seasonal temperature fluctuations is larger in the lagoons, ranging from as low as −2.1°C in the winter to over 10°C in the summer, while that in much of the rest of the Arctic Ocean does not exceed 0–4°C (Timmermans and Ladd, 2018). Salinity fluctuations are also larger in the lagoons, in some cases ranging from hypersaline in winter due to sea ice brine rejection to nearly fresh conditions in spring due to river inputs (Harris et al., 2017). The organisms inhabiting these lagoon systems must be capable of surviving rapid changes in physical and chemical conditions.

Several studies have demonstrated that organic carbon from terrestrial runoff subsidizes lagoon food webs in the Arctic (Dunton et al., 2006, 2012; Bell et al., 2016; Mohan et al., 2016; Harris et al., 2018). These subsidies likely enter food webs *via* heterotrophic bacterial and protistan communities; however, the extent to which terrestrial subsidies influence the composition of microbial communities in these lagoons remains unknown. One study in a lagoon near Barrow, Alaska, used experimental incubations to show a change in Arctic marine bacterial community composition and an increase in production in response to tundra-derived organic matter amendments (Sipler et al., 2017). Understanding how coastal microbial populations incorporate terrestrial organic matter and use terrestrially derived nutrients is paramount to refining our understanding of pathways for the integration of terrestrial carbon into coastal Arctic marine systems. A first step in achieving this is to characterize how microbial populations in terrestrially influenced Arctic waters change seasonally and in response to inputs of riverine material.

In this study, we describe seasonal variation in prokaryotic and protistan community composition in coastal lagoons of the Alaskan Arctic Ocean, and identify potential controls on microbial population dynamics, including organic matter source and prokaryotic-eukaryotic associations. This work was carried out in the context of a larger interdisciplinary study aimed at understanding how terrestrial inputs control physical (Harris et al., 2017), biogeochemical (Connelly et al., 2015; Mohan et al., 2016), and ecological (Nolan et al., 2011; Dunton et al., 2012; Harris et al., 2018) properties of lagoon ecosystems along the Alaskan Beaufort Sea coast.

## Materials and Methods

### Sample Collection

**Figure 1:**
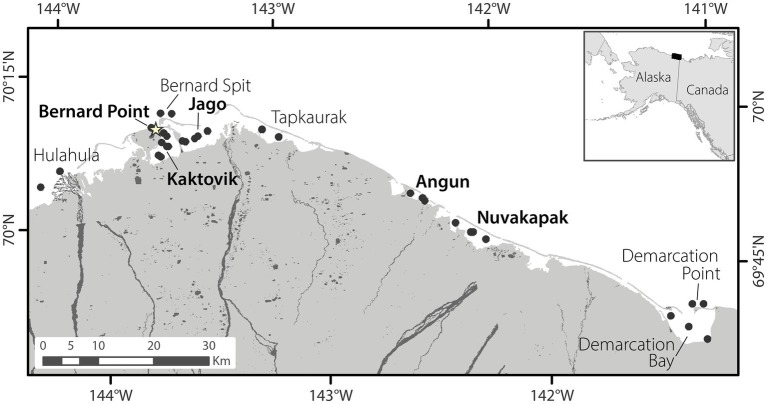
Map of the sampling region. Black circles indicate all locations from which samples were collected. Site names in bold were sampled in all seasons, while those not bolded were sampled only in August. The star indicates the location of the town of Kaktovik, Alaska.

Water samples (2 L) for microbial community analyses were collected from several sites within lagoons and outside barrier islands along the Alaskan Beaufort Sea coast in August 2011, and April, June, and August 2012 and 2013. Four lagoons, Kaktovik (KA), Jago (JA), Angun (AN), and Nuvagapak (NU), and one site outside the barrier islands near Barter Island (BP) were sampled in all three seasons (Figure 1, BP was not sampled in August 2011). Two more lagoons, Tapkaurak (TA) and Demarcation Bay (DE), and three additional sites outside the barrier islands, near the Hulahula River (HU), Bernard Spit (BE), and Demarcation Point (DP), were also sampled in August (Figure 1). Severe weather limited sample collection to KA, JA, AN, BP, and BE in August 2013. Samples were collected from one to two stations per site in April and June, and two to three stations per site in August of each year. BP had only one station in all seasons. Most sites were less than 4 m deep, with the exception of BE and DP, which were ~9–10 m deep. Samples were collected approximately 10 cm below the bottom off the ice cover in April (ice thickness 1.3–1.7 m) and from the top 2 m of the water column in June using a peristaltic pump, and by submerging hand-held sample bottles to ~0.5 m below the water surface in August. River endmembers were collected from the Canning, Jago, and HulaHula rivers in August 2011 and from Canning and Jago rivers in August 2012.

Samples were also collected for a suite of environmental measurements including particulate organic carbon (POC) and nitrogen (PON) concentrations and stable isotope ratios (POC δ^13^C and PN δ^15^N), chlorophyll *a* (Chl *a*) concentration, dissolved organic carbon and nitrogen concentrations, dissolved inorganic nitrogen (DIN = NO_3_ + NH_4_) concentrations, and oxygen stable isotope ratios of water (H_2_O-δ^18^O). Sample processing methods and measurements of particulate parameters (POC, PON, Chl *a*) are discussed in Connelly et al. (2015). Methods for dissolved parameters follow procedures described in McClelland et al. (2014). A YSI Sonde was used for measuring temperature, salinity, and dissolved oxygen from depths sampled (in addition to other depths throughout the water column). See Harris et al. (2017) for details of physical measurements and oxygen stable isotope ratios.

### Microbial Sample Processing, DNA Extraction, and Polymerase Chain Reaction Amplification

After collection, samples were kept under shade during transit back to the Arctic National Wildlife Refuge field station in Kaktovik, Alaska. Within hours of collection, 2 L of water was filtered onto a 0.22-μm Sterivex filter (Millipore) using a peristaltic pump and preserved with 1 ml of DNA extraction buffer (100 mM Tris, 100 mM NaEDTA, 100 mM phosphate buffer, 1.5 M NaCl, 1% CTAB) and kept frozen until extraction. Prior to filtration, duplicate 14-ml samples were collected from the sample bottles, fixed with glutaraldehyde (2% final concentration), and frozen for estimation of bacterial abundance using flow cytometry.

Prior to extraction, Sterivex filter cartridges were cracked open with pliers and filters were removed using an ethanol-flamed scalpel. The DNA extraction buffer from the cartridge was decanted into a sterile 2-ml microcentrifuge tube and the filter was subsequently cut into multiple pieces on a sterile cutting board and placed in the same tube. Samples were then subjected to three freeze-thaw cycles, followed by enzymatic lysis with Lysozyme (0.2 mg/ml final concentration) and Proteinase K (2 mg/ml final concentration) at 37°C for 30 min and continued digestion and lysis with the addition of SDS (1% final concentration) at 65°C for up to 2 h. Samples were then extracted two times with an equal volume of Phenol:Chloroform:Isoamyl alcohol (25:24:1) and nucleic acids were precipitated using 100% isopropanol (0.6 × volumes of the resulting supernatant) for 2 h up to overnight. Samples were then pelleted at 18,000 RCF for 30 min, rinsed, and re-pelleted two times with 70% ethanol, and dried down in a roto-evaporator. Once dry, samples were resuspended in 250 ml of nuclease-free water.

For community composition analysis, we amplified the V4 region (515F, GTGCCAGCMGCCGCGGTAA and 806R, GGACTACHVGGGTWTCTAAT) of the 16S rRNA gene for prokaryotic composition, and the V9 region (1391F, GTACACACCGCCCGTC and EukBr, TGATCCTTCTGCAGGTTCACCTAC) of the 18S rRNA gene for eukaryotic composition for sequencing on the Illumina MiSeq platform using Earth Microbiome Project protocols (http://www.earthmicrobiome.org/protocols-and-standards/16s/, but with only 30 PCR cycles). However, a known mismatch in the 16S primers with Thaumarcheaota, a dominant phylum of the marine Archaeal community, precluded us from drawing conclusions about Archaeal community composition. Each sample was amplified three times, pooled, quantified using Picogreen, and then, for each amplicon, pooled at equimolar concentrations (100 μmol each). The 16S sample pool and 18S sample pool were each cleaned using a MoBio Ultraclean PCR Clean-Up Kit and quantified using Picogreen. Amplicon pools were sequenced at Argonne National Lab (the 16S sample library composed of August 2011, and April and June 2012 samples) or the Oregon State University Center for Genome Research and Biocomputing (all 18S sample libraries and an additional 16S library from August 2012 and all 2013 samples) 2 × 150 bp paired-end reads. Gene amplicon sequences have been deposited in NCBI Sequence Read Archive (SRA) bioproject accession number PRJNA530074, under run accessions SRR8832739-SRR8833063 (16S rRNA gene) and SRR8837972-SRR8838296 (18S rRNA gene)^1^.

### Bacterial Abundance Measurements

Cell counts were performed using a BD Biosciences FACSCalibur Flow Cytometer at UMCES Horn Point Laboratory (2011 and 2012 samples) and Oregon State University (2013 samples). Single samples were counted for 2011 sites, while duplicate samples were counted and averaged for all sites after 2011. In the field, 14 ml of seawater was preserved with glutaraldehyde (2% final concentration) and frozen. In the lab, samples were thawed and 1.5-ml aliquots were stained overnight in the case of 2011 and 2012 samples with 20 μl of 1:200 SYBR Green I. The next day, samples were spiked with 15 μl (25 μl in 2013) of a sonicated beadstock created from PeakFlow Flow Cytometry Reference Beads (Life Technologies, Inc.) for internal reference. Samples from 2013 were stained and counted on the same day. Data were collected using the program CellQuest Pro (BD Biosciences) in logarithmic mode based on side scatter (SSC) and green fluorescence (FL1) with a target rate of 100–1,000 events s^−1^ for a total of 20,000 events for 2011–2012 samples and for a set period of time for 2013 samples (average 78,000 events). See Meyer et al. (2014) for additional methodological details, including how cell concentration was calculated from counted events.

### Sequence Analysis

Reads that were successfully paired using fastq-join (Aronesty, 2011) were quality filtered with an expected error rate of 0.5, dereplicated (derep_fulllength), and abundance sorted (sortbysize) using UPARSE v. 8 (fastq_filter; Edgar, 2013). Singleton sequences were removed in the latter step to prevent them from seeding clusters when clustering sequences into operational taxonomic units (OTUs). Reads were then clustered into OTUs (cluster_otus in UPARSE pipeline) at 97% similarity. A *de novo* chimera check is inherent in the cluster_otus algorithm and chimeric sequences were removed during OTU clustering. Reference-based chimera filtering was performed using UPARSE (uchime_ref) with the Gold Database^2^ as reference. Reads (including singletons) were subsequently mapped back to OTUs using UPARSE (usearch_global) and an OTU table created. Taxonomy of the representative sequences was assigned in QIIME v. 1.9 (assign_taxonomy.py; Caporaso et al., 2010) using the RDP classifier trained to the Greengenes database (v. 13.8, http://greengenes.secondgenome.com/) for 16S amplicons or the Silva database (v. 119; Quast et al., 2013; Yilmaz et al., 2014) for 18S amplicons. Any remaining singletons and OTUs occurring in only one sample were removed in QIIME (filter_otus_from_otu_table.py). Sequences identified as Archaeal, chloroplast, and mitochondrial were also removed from 16S reads. For the 18S rRNA gene library, we removed clades known to have multicellularity, as well as unclassified reads, in order to focus on protists. After these quality control steps, the average number of reads per sample was 22,326 for 16S amplicons (range 3,651–73,169 sequences per sample) and 43,093 sequences for 18S amplicons (range 6,720–103,750 sequences per sample).

### Statistical Analyses

Given recent insights that rarefying microbiome datasets may not be the best method for comparing samples (McMurdie and Holmes, 2014), we chose not to randomly subsample OTU tables for the bulk of our analyses, with the exception of alpha diversity estimates. For alpha diversity measurements, the 18S rRNA gene OTU table was rarefied to 6,700 sequences per sample, and the 16S rRNA gene OTU table to 3,650 sequences per sample. Alpha diversity was calculated as Chao1 Diversity Index to measure species richness (Chao, 1984), Simpson’s Evenness Measure (Smith and Wilson, 1996) to measure evenness, and Phylogenetic Diversity, which incorporates phylogenetic differences among species in the calculation of diversity (Faith, 1992; Caporaso et al., 2011). For beta diversity analyses, comparisons with environmental data, and indicator species analysis, OTU tables were normalized using proportional abundance of each OTU within each sample. To verify that using proportional abundance did not substantially change our conclusions compared to using OTU tables that were subsampled, we ran a subset of the analyses described in this paper using rarefied OTU tables and found no significant difference in results or conclusions.

Microbial community structure was assessed using nonmetric multidimensional scaling (NMDS) calculated using the metaMDS function in the Vegan package for R (Oksanen et al., 2019). Variability in bacterial and eukaryotic community composition among samples was calculated using Bray-Curtis dissimilarity. Permutational multivariate analysis of variance (PERMANOVA; Anderson, 2017) and Analysis of Similarity (ANOSIM; Clarke, 1993) calculated using the adonis and anosim functions in the Vegan package for R (Oksanen et al., 2019) were used to test for differences among sample groupings determined *a priori* (e.g., by season, inside versus outside of barrier islands). PERMANOVA provides a pseudo-F-ratio, a value of *p* for the group-wise tests for differences (as you would get from a standard ANOVA), and the percent of variation in the community dataset explained by the grouping. ANOSIM provides an *R* value ranging from 0 to 1 with higher values indicating stronger differences between or among groups, and a significance value for the ANOSIM *R* value based on 999 permutations.

The degree to which physico-chemical data explained the variation in bacterial and eukaryotic communities was assessed using three methods. First, a Procrustes analysis was used to compare ordinations of community and physico-chemical data (Peres-Neto and Jackson, 2001) yielding correlation and significance values. Second, envfit in the Vegan package of R was used to decipher which variables were contributing to the structure of community nonmetric multidimensional scaling ordinations by fitting vectors of significant physico-chemical variables onto community NMDS ordinations. Finally, redundancy analysis (RDA) was used to quantify the percent of variation in bacterial or eukaryotic community composition explained by the physico-chemical environmental characteristics. Bacterial and Eukaryotic OTU tables were Hellinger-transformed prior to use in the RDA. Before running the RDA, physico-chemical variables for the model were selected to reduce multicollinearity using correlation matrices. The absence of substantial multicollinearity in this subset of variables was verified using the vif.cca function available in the Vegan package for R. RDA was run using the Vegan package for R.

Indicator species analysis (Dufrêne and Legendre, 1997) was used to identify bacterial and eukaryotic taxa that significantly contributed to seasonal differences in the coastal Beaufort Sea microbial community. In order to distinguish between river indicator species and lagoon indicator species, four sample groups were used for this analysis: River, April, June, and August. The indval program in the labdsv package for R was used to run the Dufrêne-Legendre Indicator Species Analysis, and OTUs having an indicator value (IV) > 0.7 and *p* < 0.005 were considered significant indicators. Monthly indicators were then further broken down into two groups, high-abundance indicators, having an average relative abundance of greater than 0.5% of the total average population for that month, and low-abundance indicators, which were significant but had an average relative abundance of less than 0.5%. The taxonomic composition of only high-abundance indicators was further scrutinized. The relationship between the distribution of high-abundance indicators and the Beaufort Sea environment was examined using Spearman correlations, with values of *p* adjusted using the Benjamini-Hochberg correction (Benjamini and Hochberg, 1995). Correlations were calculated using the Hmisc package for R, while the calculated values of *p* were adjusted using the base R stats package.

### Co-occurrence Network Analysis

Microbial association networks were generated for each month, across all years, using CoNet (Faust et al., 2012). In order for an OTU to be included in the network it had to be present in 25–33% of the samples (April_minocc_ = 4, June_minocc_ = 5, and August_minocc_ = 14). In April and June, a percentage slightly higher than 25% was used because the number of correlations was very large and computation time was too great, preventing completion of network calculations at a minimum occurrence of 25%. Pairwise scores were computed for both Bray–Curtis similarity and Spearman correlation. Associations with a Spearman correlation above 0.7 or below −0.7 and a Bray-Curtis similarity of above 0.6 or below 0.4 were retained. For each measure and edge, 1,000 permutations (with renormalization for correlation measures) and bootstrap scores were generated, following the ReBoot routine. Values of *p* were calculated as described in Weiss et al. (2016) and measure-specific values of *p* were merged using Brown’s method. Associations were corrected using the Benjamini-Hochberg’s false discovery rate (Benjamini and Hochberg, 1995) and edges with merged values of *p* below 0.05 were retained. Edges had to be significant using both similarity measures to be kept. Network statistics were calculated in Cytoscape 3.6.1 (Smoot et al., 2011). Chord diagrams, created using the R package circlize, were used to display significant associations among the 15 most abundant taxa groups across all three networks (Gu et al., 2014).

## Results

### Environmental Conditions

**Figure 2:**
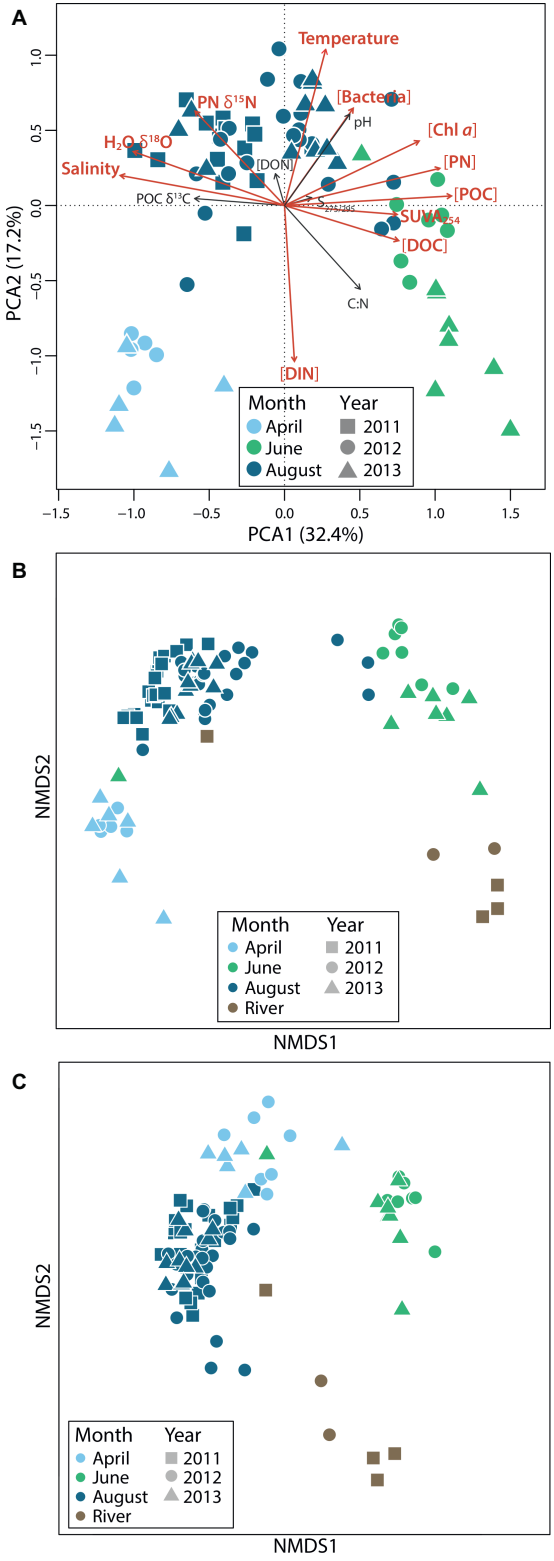
Environmental and microbial community variability across seasons. Principal component analysis of **(A)** physico-chemical data and nonmetric multidimensional scaling plots showing **(B)** bacterial and **(C)** protistan beta diversity (based on Bray-Curtis distances) highlight seasonal variation.

April waters were ice-covered and cold (average of −2°C; Harris et al., 2017), with high salinity and inorganic nutrients, and low Chl *a*, dissolved oxygen, pH, organic matter, and bacterial abundance (Figure 2A). June waters, sampled during ice break-up, were also cold but had the highest organic matter concentrations, the highest SUVA_254_ (a measure of DOC aromaticity), and the lowest salinity because of freshwater input from rivers (Table 1). August waters were warmer (average of 8.9°C), with lower concentrations of inorganic nutrients and organic matter, and higher values of H_2_O-δ^18^O, POC δ^13^C, and PN δ^15^N. Ranges of these variables fluctuated interannually, but seasonal patterns of change in coastal Beaufort Sea waters were the same from year to year.

**Table 1:** Monthly average physical and chemical properties across all lagoons.

	April (*n* = 13)	June (*n* = 15)	August (*n* = 57)
Salinity	35.6 (4.4)	5.4 (9.9)	22.5 (6.8)
Temperature (°C)	−2.0 (0.3)	2.0 (1.5)	8.9 (2.9)
DO (mg l^−1^)	12.1 (2.1)	13.4 (1.0)	10.7 (1.3)
Chl *a* (μg l^−1^)	0.042 (0.03)	2.3 (3.8)	0.38 (0.4)
pH	7.5 (0.3)	7.9 (0.3)	7.9 (0.2)
BA (× 10^8^ cells/L)	4.0 (4.5)	6.6 (2.9)	8.7 (5.5)
DOC (μmol)	107.9 (23.2)	211.3 (57.2)	109.9 (37.7)
DON (μmol)	5.4 (6.6)	6.6 (2.9)	6.4 (2.0)
DOC:DON	15.6 (11.8)	45.4 (43.5)	18.5 (7.5)
SUVA_254_	2.3 (1.1)	3.5 (0.5)	2.6 (1.0)
S_275–295_	−0.014 (0.005)	−0.014 (0.001)	−0.014 (0.004)
TDN (mg/l)	0.18 (0.09)	0.13 (0.03)	0.10 (0.02)
NO_3_ (μmol)	2.8 (2.0)	1.2 (1.6)	0.081 (0.3)
NH_4_ (μmol)	4.9 (10.5)	1.4 (1.6)	0.3 (0.7)
POC (μg l^−1^)	106.6 (119.7)	538.4 (152.3)	216.4 (75.7)
PN (μg l^−1^)	17.9 (22.5)	74.4 (19.3)	36.6 (12.9)
POC:PN	6.7 (1.3)	7.3 (1.2)	6.0 (0.9)
POC δ^13^C (‰)	−26.7 (1.3)	−28.5 (1.4)	−26.8 (2.6)
PN δ^15^N (‰)	5.2 (2.4)	4.7 (2.3)	6.5 (1.7)
H_2_O-δ^18^O	−3.7 (0.5)	−15.3 (3.2)	−6.4 (3.0)

*BA, bacterial abundance*.

*Standard deviations are given in parentheses*.

### Bacterial Diversity and Community Composition

#### Alpha Diversity

We identified 17,340 bacterial OTUs and 9,583 protistan (unicellular eukaryotes, including fungi) OTUs. For bacterial OTUs, river samples had the highest species richness and phylogenetic diversity (FDR-corrected *p* < 0.005). Evenness in river samples was not significantly different from lagoon coastal waters in April and August. Among lagoon and coastal samples, richness was highest in June (FDR-corrected *p* < 0.01, Supplementary Figure S1), phylogenetic diversity was lowest in August (FDR-corrected *p* < 0.01), and evenness was highest in August (FDR-corrected *p* < 0.01) and lowest in June (FDR-corrected *p* < 0.005). There was no interannual variability in richness and evenness in April or June, but evenness was significantly greater in August 2012 and 2013 than in 2011 (FDR-corrected *p* < 0.05, Supplementary Figure S2). Bacterial richness and evenness were the same between sites within and outside of barrier islands except in August when evenness outside the barrier islands was lower (FDR-corrected *p* = 0.0035, Supplementary Figure S3).

As with bacteria, eukaryotic species richness was greatest in rivers, but unlike bacteria the coastal eukaryotic communities had the lowest richness and phylogenetic diversity in June and the highest in April (Supplementary Figure S1). No significant differences in richness were observed among the eukaryotic communities when grouped by month or location. There was also no interannual variability in eukaryotic richness, phylogenetic diversity, or evenness except in August 2011 when richness and phylogenetic diversity values were significantly lower than later years (FDR-adjusted *p* < 0.05, Supplementary Figure S2). Eukaryotic richness and evenness were the same between sites within and outside barrier islands in June, but richness was greater outside the islands in April (FDR-adjusted *p* < 0.05, Supplementary Figure S3) and evenness was greater inside the islands in August (FDR-adjusted *p* < =0.021, Supplementary Figure S3).

#### Taxonomic Composition

**Figure 3:**
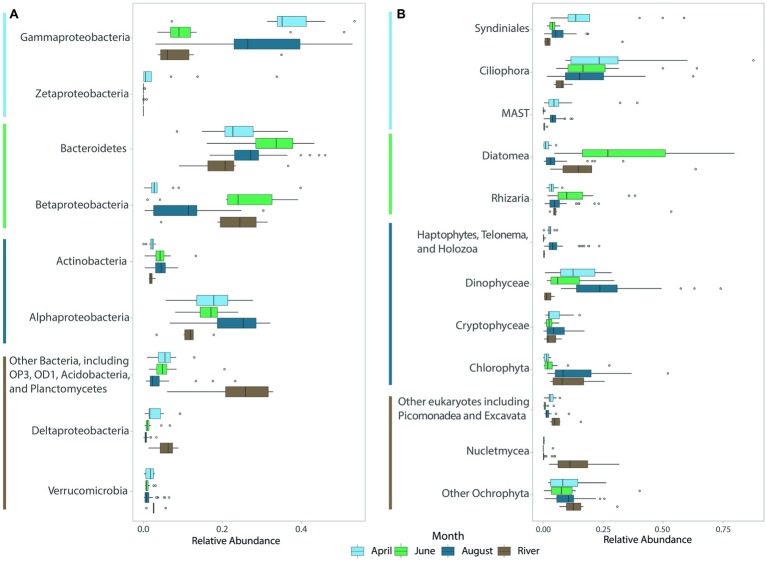
Boxplots of abundant **(A)** bacterial and **(B)** eukaryotic (protistan and fungal) groups for each month across all years sampled. River samples, though collected in August, were averaged separately. The color of the lines next to taxa names indicates the month in which each taxon or group of taxa was most abundant.

River bacterial communities were dominated by Betaproteobacteria (22% of the community on average), Bacteroidetes (21%), Gammaproteobacteria (11%), and Alphaproteobacteria (11%) (Figure 3A). Coastal bacterial communities in April and August were dominated by Gammaproteobacteria (36.3 and 29.6%, respectively), Bacteroidetes (23.7 and 27.2%), and Alphaproteobacteria (17.4% in April and 23.4% in August), but differed in the abundant members of these groups. For example, in August, Gammaproteobacteria included many members of the oligotrophic marine clades SAR86, SAR92, OM60, and OM182 groups (43% of Gammaproteobacteria), whereas in April these taxa were less abundant (20% of Gammaproteobacteria) and the Gammaproteobacteria were dominated by other members of the orders Alteromonadales and Oceanospirillales (64% of Gammaproteobacteria, Supplementary Figure S4). April communities also included a large population of iron-oxidizing Zetaproteobacteria (4.6%; Figure 3A), and a diverse community of Deltaproteobacteria including the putative chemolithoautotrophic bacteria SAR324 (Sheik et al., 2014). June coastal bacterial communities were dominated by Bacteroidetes (32.6%), and Betaproteobacteria (25%), in part reflecting riverine influence, but many members of these groups differed from those in river samples (Figure 3A). For example, Betaproteobacteria in coastal waters were dominated by the marine genus *Polaromonas* (48% of Betaproteobacteria), while river samples were dominated by other Burkholderiales (64% of Betaproteobacteria, Supplementary Figure S5). Also, Bacteroidetes in June were dominated by members of the class Flavobacteriia (80% of Bacteroidetes), while this class made up only 49% the Bacteroidetes community in river samples (Supplementary Figure S6).

River protistan and fungal communities were dominated by Diatoms (20.7%), with substantial contributions from other Ochrophytes (14.7%; especially Ochromonas sp. CCMP1899), Nucletmycea (13.7%), Rhizaria (12.5%), and Chlorophytes (11.1%; Figure 3B). April eukaryotic communities were dominated by Ciliophora (27.8%) and Syndiniales (20.1%), with Dinophyceae (13.8%), non-Diatom Ochrophytes (10.6%), and marine stramenopiles (9.3%) also contributing to ice-covered eukaryotic populations (Figure 3B). June eukaryotic communities were dominated by Diatoms (33.3%), Ciliophora (21.6%), Dinophyceae (10%), and Rhizaria (13.3%). River and lagoon eukaryotic communities in June were both dominated by Diatoms, but the dominant taxa differed (rivers: *Fragilaria* sp. (68%); lagoons: *Chaetoceros* (59%) and *Skeletonema* spp. (30%); Supplementary Figure S7). Eukaryotic communities transitioned from diatom-dominated during ice break-up in June to dinoflagellate-dominated in August (24.8%) when communities also included high proportions of Ciliophora (17.4%), Chlorophyta (13.1%), and non-Diatom Ochrophytes (10%, Figure 3B). Across all seasons, Spirotrichea was the dominant group of ciliates observed in coastal samples, with Heterotrichea also abundant (5%) in April. Gymnodiniphycidae was the dominant dinoflagellate taxa (Supplementary Figure S8).

#### Beta Diversity

With respect to beta diversity, both bacterial and eukaryotic communities differed seasonally, and all coastal communities differed from river communities (Figures 2B,C). We found that 43% of the variance in bacterial communities and 27% of the variance in eukaryotic communities was accounted for by seasonal coastal and river group differences (*p* = 0.001, using PERMANOVA). Pairwise seasonal differences, as quantified using ANOSIM tests, were greater among eukaryotic communities (0.62–0.97 for eukaryotes and 0.4–0.96 for bacteria, *p* = 0.001) with the exception of April–June comparisons, which had a higher ANOSIM *R* value for bacteria than for eukaryotes (0.93 vs. 0.88, *p* = 0.001). In total, 1,207 bacterial OTUs and 712 eukaryotic OTUs were shared among all coastal microbial communities. Among coastal communities, June had the greatest number of unique bacterial OTUs (49.8%) but the fewest unique eukaryotic OTUs (38.9%). August had the most unique eukaryotic OTUs (55%). June coastal communities shared the greatest percentage of OTUs with river communities among all coastal water-river water comparisons (38.8% for bacteria and 50.9% for eukaryotes). August communities also shared a substantial percentage of their OTUs with river communities (34.1% for bacteria and 31.7% for eukaryotes), but April samples shared 20% or less of their OTUs with river communities.

#### Indicator Species

**Figure 4:**
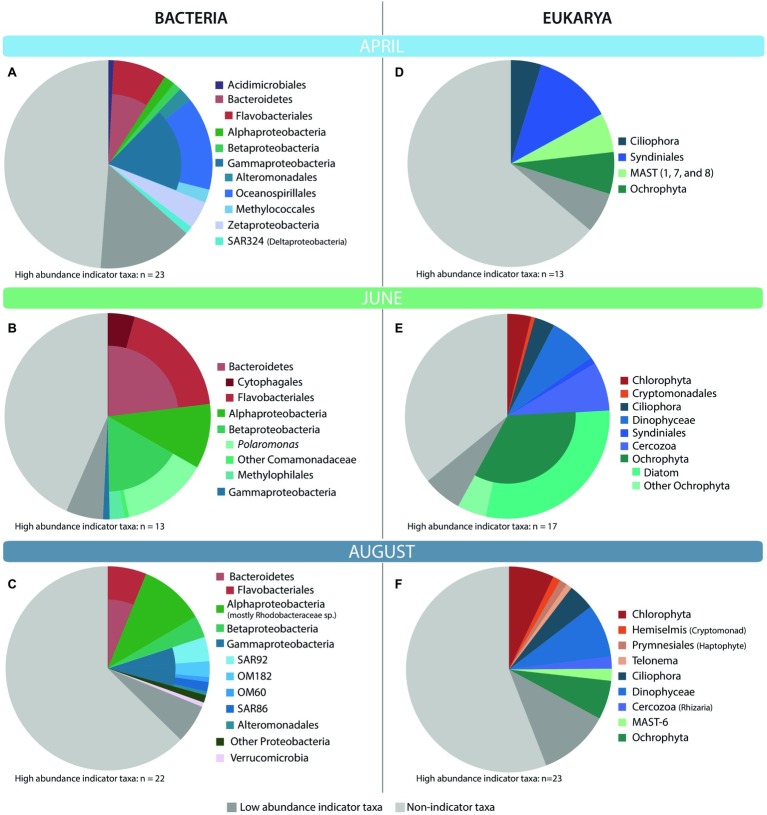
Taxonomic affiliation of top bacterial **(A–C)** and eukaryotic **(D–F)** high-abundance indicator OTUs for each month. High-abundance indicator OTUs are those OTUs that had an indicator value of >0.7, *p* < 0.001, and made up at least 0.5% of the community for the month in which they were an indicator. The monthly average relative abundance of these indicator OTUs is shown relative to the average relative abundance of non-indicator and low-abundance (<0.5%) indicator taxa.

Indicator species analysis was used to determine which OTUs significantly contributed to differences among seasons. We focused on OTUs with indicator values greater than 0.7, values of *p* less than 0.001, and average relative abundance greater than 0.5% of the average community composition for the month for which the OTU was an indicator. In April, 23 bacterial indicator taxa made up 36% of communities in these ice-covered waters (Figure 4A). Many of these indicator taxa belonged to the Gammaproteobacteria order Oceanospirillales and the Bacteroidetes order Flavobacteriales. April indicator taxa also included members of the methanotrophic order Methylococcales, and several chemolithoautotrophic taxa including iron-oxidizing Zetaproteobacteria and sulfur-oxidizing SAR324. In June, 13 indicator taxa composed 50% of communities in these highly productive, lower salinity waters (Figure 4B). Most of these taxa belonged to the Bacteroidetes phylum, and to the Betaproteobacteria genus *Polaromonas* and methylotrophic order Methylophilales. June indicators also included Alphaproteobacteria related to *Loktanella* sp. In August, 22 indicator taxa made up 31% of the bacterial communities in these late summer, nutrient-poor waters (Figure 4C). Many of these indicators belonged to the Alphaproteobacteria family Rhodobacteraceae, including *Phaeobacter* and *Octadecabacter* spp. August indicators also included Gammaproteobacteria from oligotrophic marine clades (OM60, OM182, SAR86, and SAR92).

As with the bacteria, a small number of eukaryotic indicator taxa made up a large fraction of the average April, June, and August communities (Figure 4). In April, 13 eukaryotic indicator taxa from four phyla made up 31% of the April eukaryotic community (Figure 4D) and included several OTUs closely related to the parasitic order Syndiniales, and several marine stramenopiles (MAST) belonging to groups 1, 7, and 8. In June, a more diverse set of 17 indicator taxa made up 58% of the eukaryotic community (Figure 4E). June indicators were dominated by several diatoms closely related to *Chaetoceros*, *Skeletonema*, and *Melosira* sp., but also included a diverse community of taxa from the class Dinophyceae (dinoflagellates), phyla Ciliophora (ciliates), Chlorophyta (green algae), and Cercozoa. In August, an even broader array of indicators was observed, with 23 OTUs representing 32% of the eukaryotic community (Figure 4F). August indicators were dominated by Chlorophyta, Dinophyceae, and Ochrophyta OTUs, but included other taxa such as haptophytes, cryptophytes, and ciliates. While diatoms made up the bulk of the Ochrophyta indicators in June, this was not the case in August, when Ochrophyta indicators instead belonged to the Dictyochophyceae, Chrysophyceae, and Pelagophyceae.

### Environmental Drivers of Coastal Beaufort Sea Microbial Communities

Several methods were used to investigate relationships between physico-chemical parameters and microbial community composition. First, Procrustes analysis showed that both bacterial and eukaryotic community composition were significantly correlated with variation in Beaufort Sea lagoon environmental conditions (Corr_BAC_ = 0.7278, Corr_EUK_ = 0.5923, sig = 0.001). Second, physico-chemical vectors that correlated significantly with bacterial and eukaryotic NMDS ordinations were overlain onto NMDS plots to determine the environmental gradient that correlated with the variations in community structure (Supplementary Figure S10). For the bacterial community, the first NMDS axis was negatively correlated with salinity and POC δ^13^C (*r*^2^ < −0.7) and positively correlated with SUVA_254_, POC, DOC, and Chl *a* suggesting that separation of June communities from August and April along this NMDS axis represents gradients in terrestrial input and productivity. The second NMDS axis was strongly correlated with temperature (*r*^2^ < −0.9) and negatively correlated with nitrate and ammonium (*r*^2^ > 0.9, Supplementary Figure S10A), suggesting that separation of August from April communities is driven by seasonal changes in temperature and nutrients. Finally, redundancy analysis was used to quantify the amount of variation explained by these physico-chemical variables (Supplementary Figure S11). Approximately 70% of the variation in bacterial community composition could be explained by the Beaufort Sea environment.

For the eukaryotic community, the orientation of samples on the NMDS was rotated slightly relative to the bacterial NMDS, but the major trends were essentially the same. April and August communities were separated from June communities along gradients in terrestrial input and productivity (Supplementary Figure S10B). The C:N ratio of particulate organic matter (POM) and concentrations of DOC, nitrate, and ammonium were all strongly correlated this first NMDS axis (*r*^2^ > 0.7), suggesting that the August and April communities existed in waters with more degraded organic matter and lower nutrients than June communities. Like bacterial communities, April and August eukaryotic communities separated along a temperature and nutrient gradient, but productivity (e.g., Chl *a*) and organic matter source components (SUVA_254_, Chl *a*, Salinity, and POC δ^13^C) were also important correlates with the second NMDS axis for the eukaryotic ordination (Supplementary Figure S10B). Finally, redundancy analysis showed that approximately 55% of the variation in eukaryotic community composition could be explained by the Beaufort Sea environment (Supplementary Figure S11).

**Figure 5:**
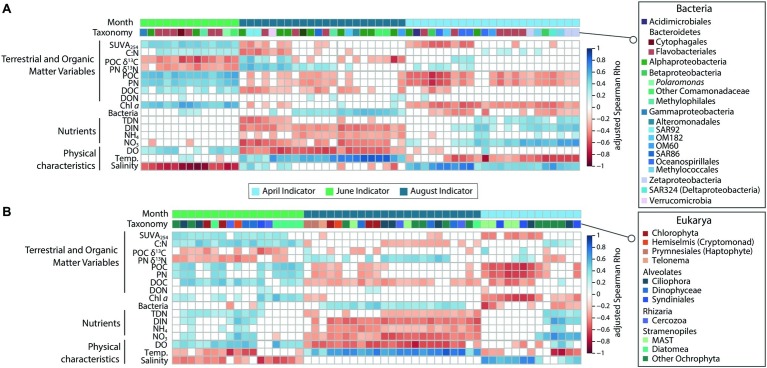
Heatmap showing significant (FDR-corrected) Spearman correlations between top indicator OTUs and physico-chemical variables for **(A)** Bacteria and **(B)** Protists. Both the month and taxonomic affiliation of the indicator OTU are indicated above the heatmap.

Correlations between high-abundance indicator taxa and measured physico-chemical variables reflected those of the whole communities (Figure 5). April indicator taxa in ice-covered waters correlated with less productive and cold, nutrient-rich conditions (negative correlations POC, PN, Chl *a*, bacterial abundance; positive correlations with salinity and inorganic nutrients). This was particularly true for the eukaryotic MAST and Syndiniales OTUs, and several bacterial indicator taxa known to use diverse metabolic strategies for survival (e.g., Methylococcales, Zetaproteobacteria, and SAR324; Figure 5). During ice break-up, June indicator OTUs correlated with conditions representing riverine input (positive correlations with SUVA_254_, C:N, POC, PN, DOC, Dissolved Oxygen, and Chl *a*; negative correlations with POC δ^13^C and PN δ^15^N and Salinity; Figure 5). August indicators correlated with conditions representing post-bloom, nutrient-depleted conditions (positive correlations with bacterial abundance and temperature; negative correlations with dissolved oxygen, nutrients, DOC, POC, and PN; Figure 5).

### Microbial Co-occurrence Network Properties

#### Network Structure

Co-occurrence networks for the three seasons were strikingly different in size and topology (Table 2). The percentage of bacterial and eukaryotic OTUs retained in these networks after frequency filtering and subsequent co-occurrence analyses varied from 7 to 36% (Supplementary Table S1), causing the networks to vary substantially in size. Among the taxa retained in these networks, significant combinations represented 1.4–14.9% of the possible combinations (Supplementary Table S1), and average path length, or the number of nodes needed to link two nodes was short, ranging from 3.1 to 4.3 (Table 2). Network diameter, or the longest distance in a network, ranged from 10 edges (June) to 13 edges (August). Network density, a normalized measure for the average connectivity within a network, was the highest in June (0.057) and lowest in August (0.014). The average node degree, or the average number of connections for each node, was very high in June (111) compared to April (33) and August (9). Overall network complexity, as estimated by connectance (the fraction of all possible links that are realized in a network; Williams et al., 2002), was highest in June (0.0282) and lowest in August (0.0071) (Table 2). The number of connected components, or a set of nodes in the network graph for which there is always an interconnecting path (Corel et al., 2016), was the lowest in April (5) and highest in August (36, Table 2). If a network has only one connected component, all nodes can be linked to any other node in the network either directly or indirectly. The presence of more than one connected component indicates that some groups of nodes are segregated from the main network, not significantly correlated with any nodes in that graph.

**Table 2:** Network statistics, calculated in Cytoscape v. 3.6.1.

Network property	April	June	August
# Nodes (S)	1,272	1966	662
# Edges (L)	21,122	109,143	3,121
# Positive edges	18,601	107,836	2,650
# Negative edges	2,521	1936	471
Link density (L/S)	16.61	55.52	4.71
Connectance (L/S^2^)	0.0131	0.0282	0.0071
Ave. degree	33.2	111	9.4
Network diameter	12	10	13
Graph density	0.026	0.057	0.014
Network centralization	0.146	0.222	0.104
Connected components	5	6	35
Clustering coefficient	0.363	0.441	0.385
Average path length	3.929	3.097	4.27

Indicator taxa were most abundant in their corresponding seasonal networks, and river indicators were most abundant in the June network (Supplementary Figure S12). In all cases, these indicator taxa were outnumbered by non-indicator taxa, but in most cases indicator taxa had higher average node degrees than non-indicator taxa (Supplementary Figure S13), indicating that they were more highly connected within the networks. This was the case for seasonal indicators in the April and August networks, and for river indicator taxa in the June network underscoring the importance of river microbes in spring surface waters in these coastal lagoons.

#### Network Composition

Taxa that were abundant during each season were also abundant in corresponding seasonal networks (Supplementary Figure S14). The April and August networks shared many of the same abundant taxa (Supplementary Figure S16) including bacterial taxa Rhodobacterales, Alteromonadales, Oceanospirillales, and Flavobacteriales, and eukaryotic taxa Rhizaria, Diatomea, Ciliophora, Syndiniales, and Dinophyceae. The June network included some of the same abundant eukaryotic taxa with the addition of Chrysophyceae, but featured a different set of bacterial taxa including Legionellales and Actinobacteria (Supplementary Figure S16).

**Figure 6:**
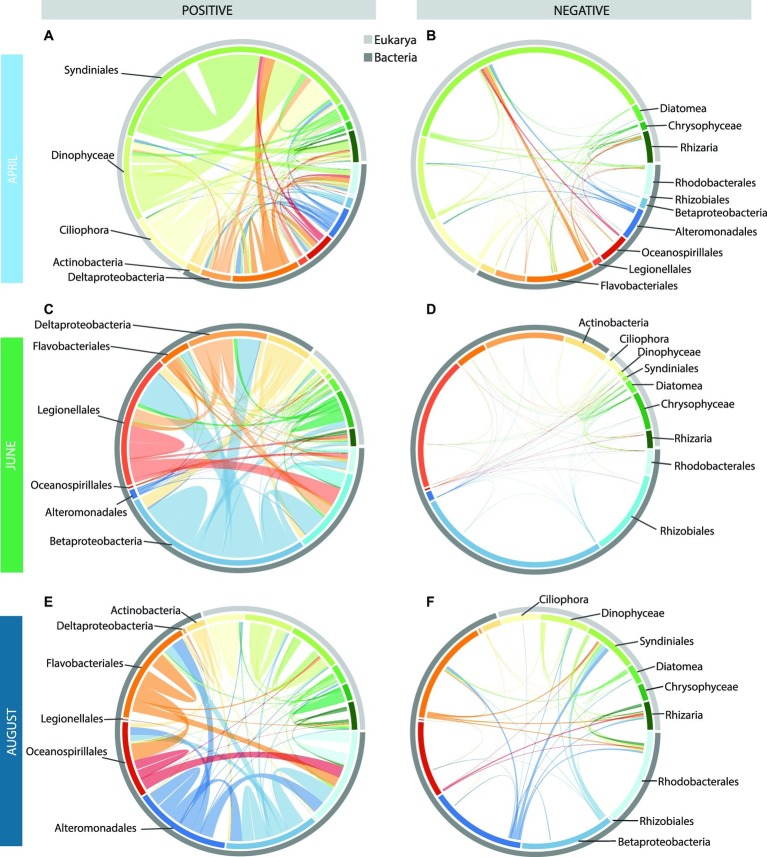
Chord diagrams showing the positive and negative correlations among the top 15 taxa in the monthly co-occurrence networks [April: **(A,B)**, June: **(C,D)**, and August: **(E,F)**]. The inner circle shows the breakdown of how the correlations within each month are distributed among these 15 taxa, with the outer circle showing the domains to which these taxa belong. The width of the bar is proportional to the number of correlations (positive and negative) for each taxon with the other 14 taxa. The arcs drawn between bars (i.e., taxa) are proportional to the number of positive (left) or negative (right) correlations between these two taxa. Arcs that remain within a bar denote significant correlations among OTUs within that taxon.

All three networks were dominated by positive edges (indicating co-presence), with far fewer negative edges (indicating mutual exclusion; Table 1). Also, organism–organism associations were far more abundant than those between organisms and environmental variables (Supplementary Figure S17). The June network was dominated by co-occurrences between prokaryotes (Figure 6), while April and August networks had a more even distribution of bacteria-bacteria (Bac-Bac), bacteria-eukaryote (Bac-Euk), and eukaryote-eukaryote (Euk-Euk) associations (Supplementary Figure S18).

We examined the positive and negative correlations among the 10 most abundant taxonomic groups within each network, which represented a total of 15 different groups (Figure 6). In April, Euk-Euk edges were dominated by Syndiniales taxa, including marine alveolate (MALV) Groups I and II and *Amoebophrya*. These taxa correlated most frequently with themselves, with dinoflagellates including Gymnodiniphycidae and Peridiniphycidae, and with ciliates, including Oligotrichia and Choreotrichia (Figure 6). Syndiniales also co-occurred with more bacterial taxa than other abundant eukaryotic taxa, and were positively correlated with Flavobacteriales and Deltaproteobacteria.

There were far fewer Euk-Euk and Bac-Euk edges than Bac-Bac edges in June, and the most abundant eukaryote in the June network, Chrysophyceae, was not the most abundant eukaryotic taxa in June samples (i.e., Diatomea). Chrysophyceae correlated mostly with themselves but also had significant correlations with Betaproteobacteria, Deltaproteobacteria, and Legionellales (Figure 6). By comparison, Diatomea had far fewer correlations within the network. The June network was dominated by Bac-Bac edges involving Betaproteobacteria, Legionellales, and Rhizobiales, all of which are commonly associated with freshwater and brackish environments (Figure 6). Deltaproteobacteria also had a high number of edges in June networks.

The August network featured many of the same taxonomic groups as the April network, but the connections among the nodes were different. Most Euk-Euk associations were positive correlations among Syndiniales and other dinoflagellates. By comparison, correlations involving ciliates were less frequent. Many Bac-Euk associations were negative, particularly those involving protist groups Syndiniales and Rhizaria and bacterial groups Alteromonadales, Flavobacteriales, Betaproteobacteria, and Rhodobacterales (Figure 6). In contrast, most Bac-Bac associations in the August network were positive.

The most connected taxa in the three networks were not always the most abundant taxa, suggesting that, in many cases, more abundant taxa may not require mutualistic interactions to thrive and can become abundant without the “help” of other microbial taxa, while the reverse may be true for the less abundant but highly connected taxa (Supplementary Figure S15). In the April network, the highest node degrees were associated with relatively rare bacterial OTUs related to the gammaproteobacterium HTCC2188, Thiotrichales, and Gemmatimonadetes, and eukaryotic taxa *Developayella* and MAST-3 OTUs, a single *Goniomonas* OTU and a *Pirsonia* OTU (Supplementary Figure S19). Similarly, in the June network, taxa with the highest node degrees were relatively rare OTUs from the Enterobacteriales, WS3, and SR1 taxa, all with average node degrees >300 (Supplementary Figure S19). However, OTUs representing the abundant taxa Rhizobiales and Betaproteobacteria also had high average node degrees (>200). In the August network, many of the abundant taxa had high average node degrees, including Oceanospirillales and Alteromonadales (Supplementary Figure S19). Also, in August, several taxa with high average node degrees featured a large fraction of negative correlations including the bacteria Saprospirales, and eukaryotes MAST-9 and *Palpitomonas* (Supplementary Figure S19).

## Discussion

Coastal waters along the North Slope of Alaska are important feeding and breeding grounds for many species of migratory birds (Johnson et al., 2007; Taylor et al., 2010) and fish, such as Arctic char and Arctic cod (Craig, 1984) that are critical to native subsistence fisheries (Dunton et al., 2012). Maintenance of healthy lagoon and coastal ecosystems is crucial to sustaining these higher trophic levels. The base of food webs in these ecosystems is occupied by several interacting and species-rich microbial communities that perform many important ecosystem services, including organic matter degradation, nutrient regeneration, and carbon fixation *via* photosynthesis. In coastal systems, these communities provide a critical pathway for the incorporation of terrestrial organic matter and nutrients into estuarine and marine food webs (e.g., Carlsson et al., 1993; McCallister et al., 2004), especially in the Arctic, where terrestrial inputs are high (Whitefield et al., 2015). In the Beaufort coastal lagoons, microbial communities must maintain ecosystem functions despite huge seasonal changes in environmental conditions. This study demonstrates that microbial communities in these lagoons respond to seasonal changes through annually repeating seasonal shifts in species composition of both prokaryotic and microbial eukaryotic communities.

### Terrestrial Subsidies

Previous studies have shown that terrestrial inputs of organic matter help fuel food webs along the Alaska Beaufort Sea coast (Dunton et al., 2006, 2012; Harris et al., 2018); yet, no studies to this point have characterized relationships between the microbial communities living within these coastal waters and the organic matter inputs to them. Characterized by relatively low concentrations of POC, PON, and pigments, especially Chl *a* (Connelly et al., 2015), April waters in these coastal lagoons had a low contribution of phototrophic microbial taxa. The suspended organic material present was highly processed, with high ratios of phaeopigments to Chl *a* and elevated saturated fatty acid proportions (Connelly et al., 2015), suggestive of a heterotroph-dominated system. Indeed we show that April communities were dominated by high proportions of small heterotrophs (e.g., MAST) and parasitic Syndiniales clades in eukaryotic communities (Figure 2B). Furthermore, the refractory nature and low concentrations of organic matter favored relatively high proportions of chemoautotrophs in bacterial communities (Figure 2A, Supplementary Figures S4, S5). OTUs belonging to family Oceanospirillaceae, members of which have been implicated in hydrocarbon degradation (Satomi and Fujii, 2014), were also in high relative abundance in April. Arctic peat contains hydrocarbons (Yunker et al., 1993) and aromatics that likely contribute to the DOM in these coastal waters, as observed further west in the coastal Chukchi Sea (Sipler et al., 2017). The ability to degrade what is commonly considered more refractory components of organic matter (Yunker et al., 2002) may give members of Oceanospirillaceae a competitive advantage at the end of winter, after all of the fresh phytoplankton-derived organic matter has been degraded.

By June, the peak of the spring freshet had passed and ice break-up was well underway. POM analyses pointed to a much more productive system characterized by carbon inputs from both terrestrial sources and autochthonous phytoplankton (Connelly et al., 2015). Eukaryotic microbial communities were dominated by diatoms, and bacterial communities by a mixture of freshwater bacteria and a distinct estuarine community that presumably grew to dominate these communities in each year of the study (Figure 3B). Bacteroidetes, including Cyclobacteriaceae and *Flavobacterium* spp., and Betaproteobacteria, particularly *Polaromonas* sp. were abundant in the June surface waters of the lagoons. Cyclobacteriaceae and *Flavobacterium* sequences were found to be enriched in low-salinity waters of the Columbia River estuary in Oregon and generally showed tolerance to a wide range of salinities (Smith et al., 2017). *Polaromonas* is a euryhaline bacterial taxa that can survive across a wide range of salinities (Veillette et al., 2011). Interestingly, this taxa was observed to be enriched in sea ice brackish brines (salinity 2.4–9.6) in the central Arctic Ocean but not in the surface seawater below the sea ice (salinity 33.3–34.9), which was thought to indicate that they were unable to survive the salinity shock during brine rejection (Fernández-Gómez et al., 2019). However, closer to the coast we observed that *Polaromonas* appears to survive this transit from sea ice into surface seawater; perhaps lower salinity surface waters resulting from river input coincident with sea ice melt provide a refuge for these taxa.

By the middle of the open-water period in August, POM was characterized by elevated proportions of terrestrial and dinoflagellate fatty acids relative to those of diatoms (Connelly et al., 2015), which was validated by a shift from a diatom-dominated community in June to a dinoflagellate-dominated community in August (Figure 3B, discussed in detail below). Coincident with these changes in OM source and decreased inorganic nutrient concentrations, the bacterial community came to resemble a typical coastal ocean community, becoming enriched in bacterial clades commonly considered to be oligotrophic, including SAR86, SAR92, and OM182 (Figure 4). Many of these clades were also present in April but in lower proportions. SAR92 and OM182 belong to the oligotrophic marine Gammaproteobacteria (OMG), while SAR86 is more distantly related and possesses an even more streamlined genome (Spring et al., 2015). OM182 and SAR86 were observed to become more abundant in late summer and fall in brackish waters of the Baltic Sea (Hugerth et al., 2015), aligned with an oligotrophic lifestyle. SAR92 is common in coastal waters at high (Ghiglione et al., 2012) and lower latitudes (Teeling et al., 2012), often in association with or just following phytoplankton blooms. Since Chl *a* was lower in August than in June, it appears that SAR92 can also persist in coastal waters of the Beaufort Sea well after peak primary production. Altogether, these parallels between our study and Connelly et al. (2015), coupled with the high percentage (55–70%) of community variation explained by physico-chemical measurements, suggest strong linkages between organic matter source and microbial community composition, and are consistent with similar seasonal changes in POM pigments and phytoplankton communities observed further east near the Mackenzie River plume (Morata et al., 2008).

### Photosynthetic Protists

In much of the Arctic Ocean, diatoms are the most abundant primary producers in spring (Figure 5), while smaller picoeukaryotes dominate the photoautotroph community the remainder of the year (Lovejoy et al., 2011; Marquardt et al., 2016). This also occurs in the Beaufort coastal lagoons in June despite significant river influence and lower salinity. *Chaetoceros* and *Thalassiosira*, two abundant taxa in June, are dominant diatoms in under-ice blooms on the Chukchi Shelf (Arrigo et al., 2012) and in pelagic spring blooms across the Arctic (Poulin et al., 2011), including in the Beaufort Sea (Balzano et al., 2012b). *Melosira* and *Navicula*, also abundant in June, are common sea-ice associated diatom taxa (Booth and Horner, 1997; Poulin et al., 2011) that are thought to seed pelagic phytoplankton communities in spring (Michel et al., 1993; Hardge et al., 2017). In the Beaufort coastal lagoons, *Melosira* was only abundant in June, suggestive of a sea-ice source, but *Navicula* was present in all seasons demonstrating that, while sea-ice may be a source for *Navicula*, members of this genus persist in the water column and contribute to the pelagic phytoplankton community (Hardge et al., 2017). Other notable primary producers in spring included the chlorophytes *Carteria*, *Chlamydomonas*, and chrysophyte *Ochromonas* which are commonly considered to be freshwater and snow genera, but their presence has been reported in Arctic coastal waters influenced by the Mackenzie River (Balzano et al., 2012a) and in sea ice and melt ponds elsewhere in the Arctic (Kilias et al., 2014). Given that members of these genera appear to survive across a wide range of salinities, these euryhaline phototrophs may become increasingly important in coastal Arctic waters, and across the Arctic as a whole, with the forecasted freshening of the Arctic Ocean (McPhee et al., 2009; Morison et al., 2012).

As spring progressed into summer, the composition of the primary producers shifted from large cells (diatoms) to smaller picophytoplankton, predominately prasinophytes *Micromonas* and *Bathycoccus* (Supplementary Figure S9). *Micromonas* was dominant in 2011 (average 11% vs. 0.9% for *Bathycoccus*) when summertime waters were relatively cold (7.9°C) and salty (27.5 PSU). *Bathycoccus* was dominant in 2012 and 2013 (4.7 and 6.5% vs. 0.6 and 0.5% for *Micromonas*) when waters were warmer and fresher (9–11.2°C, 19.6–22.1 PSU). *Micromonas* is commonly thought to be the most abundant Arctic prasinophyte (Terrado et al., 2013), but *Bathycoccus* was more abundant on the river-influenced Mackenzie Shelf (Monier et al., 2015) and during polar sunset and polar night in the Amundsen Gulf Region (Joli et al., 2017), possibly due to differences in low-light survival strategies. In both cases, light, grazing, and nutrients were hypothesized to drive this taxonomic shift. Both *Micromonas* and *Bathycoccus* have been shown to be capable of osmotrophy (Hernández-Ruiz et al., 2018), but *Bathycoccus* appears to have a stronger preference for amino acids as a carbon source relative to bicarbonate, suggesting that *Bathycoccus* is particularly adapted to organic matter utilization. Low-light adaptation and the ability to consume organic matter may explain the success of *Bathycoccus* in 2012 and 2013, when river input and organic matter concentrations were higher.

Under the ice in April, photoautotrophs were much less abundant, and consisted mainly of the prasinophytes discussed above, stramenopiles related to Bolidophyceae (2.7%) and dictyochophyte Pedinellales (3.4%), which was also abundant in August (4.1%). Bolidophyceae and Pedinellales have been observed elsewhere in the Beaufort Sea (Balzano et al., 2012a; Terrado et al., 2013), under the ice in the Central Arctic Ocean (Pedinellales only; Hardge et al., 2017), and in Canadian High Arctic sea ice (Piwosz et al., 2013). Seventeen percent of the bolidophyte cells investigated from sea ice were found to have at least one bacterium in their food vacuole (Piwosz et al., 2013) and thus their presence under ice may be sustained through heterotrophy rather than photosynthesis.

### Chemolithoautotrophs and Methylotrophs

Ice-covered waters were dominated by bacterial taxa known to thrive in low-organic matter conditions, such as chemolithoautotrophs including Zetaproteobacteria (4.6%), Deltaproteobacteria clade SAR324 (1.3%), and methylotrophs including Methylococcales (2%) (Figure 3). Zetaproteobacteria (4.6%) are mat-forming Fe(II) oxidizers that are closely related to the chemotrophic iron-oxidizing genus *Mariprofundus* (Singer et al., 2011). Their presence in April waters was consistent with the orange tint observed on several April sample filters, but was surprising given that this is, to our knowledge, the first evidence of this microbial taxa in coastal Arctic waters. Zetaproteobacteria have been observed in iron-rich hydrothermal vents of the Loihi Seamount (McAllister et al., 2011), and coastal waters in Maine, USA (McBeth et al., 2011). Our study extends their distribution to include the coastal Arctic Ocean. These lagoons receive large pulses of iron during the spring snow melt (Rember and Trefry, 2004), and iron concentration in Arctic freshwaters increases through spring and summer (Pokrovsky et al., 2016) and may be enhanced by permafrost thaw (Barker et al., 2014).

SAR324 (1.3%) have genes for sulfur and alkane oxidation and have the capacity to degrade short-chain fatty acids, among other metabolic strategies (Sheik et al., 2014). Interestingly, Connelly et al. (2015) observed the highest proportional abundance of short-chain fatty acids in April waters compared to June or August. SAR324 were also found to be proportionally more abundant in surface waters under-ice than in open waters off Point Barrow (Sipler et al., 2017) and were shown to be important in nitrogen cycling in the winter (Connelly et al., 2014). Chemoautotrophic production under-ice may help sustain biological communities during the long winter, as it is thought to in other continually ice-covered systems (Boyd et al., 2014; Vick-Majors et al., 2016).

Methylococcales are exclusively methylotrophs and type I methanotrophs that have been observed to thrive in association with iron-oxidizing microbial mats in freshwater systems (Quaiser et al., 2014). Our data suggest that iron-oxidizing and methane-oxidizing bacteria may also live in close association in iron-rich, coastal marine waters. Methane is present in shallow sediments throughout the Beaufort Sea shelf (Coffin et al., 2013), and dissolved methane is highly concentrated in Beaufort Sea water (Lorenson et al., 2016), particularly in shallow waters. This methane is mainly generated by microbial degradation of organic matter (Lorenson et al., 2016), but may also arise from permafrost-associated methane gas hydrates (Shakhova et al., 2017), which are present throughout the Beaufort shelf region (Riedel et al., 2014). We did not measure methane concentrations in our water samples, but wintertime under-ice methane concentrations to the west of our sample region were 3–28 times greater than in summer (Kvenvolden et al., 1993). While both of the iron- and methane-oxidizing taxa in our samples are aerobic, they prefer to live at oxic-anoxic interfaces to allow for the presence of both oxygen and reduced electron donors, which may have been available given the presence of low oxygen levels in some lagoons in April (Connelly et al., 2015). Overall, the presence of these bacterial functional groups suggests that iron, methane, nitrogen, and sulfur cycling become relatively important under the ice in these coastal lagoons as more labile organic matter is progressively depleted through the long Arctic winter.

### Parasites

Heterotrophic protists play an important part in marine food webs as grazers of phytoplankton and bacterioplankton, and as food for zooplankton. In the Central Arctic Ocean, their biomass can rival or exceed that of phototrophic protists (Sherr et al., 1997). Heterotrophic and parasitic protists were relatively abundant in the Beaufort coastal lagoons in all seasons, but were particularly dominant in April when sea ice and snow attenuated light penetration into surface waters, limiting the abundance of photosynthetic protists (Figure 3). Thus, heterotrophy and parasitism likely dominated the protistan lifestyle in April waters. This is corroborated by the presence of abundant sequences related to ciliates, heterotrophic dinoflagellates, parasitic Syndiniales, and MAST taxa in the under-ice community (Figure 3). Heterotrophic protists were relatively less abundant in spring, but became dominant again later in summer, following the spring bloom and depletion of macronutrients (Table 1).

Syndiniales, including the MALVs, are a globally distributed parasitic group within the Alveolates (Guillou et al., 2008; de Vargas et al., 2015), that constitute a substantial component of the global marine interactome (Lima-Mendez et al., 2015), and are generally considered to have a broad host range from other protists to metazoans (Guillou et al., 2008). We observed clear seasonality in Syndiniales, especially Groups I and II, with the greatest relative abundance under ice in April (20%), and lower relative abundance in June (4%) and August (6.3%). Syndiniales followed a similar abundance pattern in Franklin Bay further east in the Beaufort Sea (Terrado et al., 2009), in a high-Arctic Fjord (Marquardt et al., 2016), and in Antarctic waters (Cleary and Durbin, 2016). Oxygen may influence the distribution of these two groups in the water column, with Group I preferring low-oxygen waters or sediments and Group II preferring oxygenated waters (Guillou et al., 2008), although both groups were abundant in suboxic and anoxic fjord waters in British Columbia (Torres-Beltrán et al., 2018). Low dissolved oxygen was measured in some of the lagoons under the ice (Connelly et al., 2015), yet Group II were the most abundant Syndiniales in this season, which further suggests that oxygen is not the only driver of their distribution and that other factors such as host availability and host stress under winter conditions (Cleary and Durbin, 2016) control the abundance and diversity of Syndiniales.

### Grazers

Heterotrophic flagellates like marine stramenopiles are ubiquitous in the global ocean (Lovejoy et al., 2006; de Vargas et al., 2015) and, as bacterivores, represent important links in marine microbial food webs, transferring carbon from bacteria to higher trophic levels like zooplankton (Monier et al., 2013; Worden et al., 2015). More abundant in the lagoons during less productive months, MAST clades MAST-1 and MAST-6 had the highest relative abundances in our dataset with MAST-1A and MAST-1C most abundant in April (6% of 18S rRNA genes). These clades were also found to be abundant in near-ice or under-ice stations along a transect from the Labrador Sea west to the Beaufort Sea (Thaler and Lovejoy, 2013). In August, MAST-6 was the most abundant MAST clade (2.2%). This clade has rarely been reported in the Arctic, but that is likely due to the fact that it is missed by the PCR primer set commonly used to assess protist diversity in Arctic waters (e.g., Thaler and Lovejoy, 2015). Using CARD-FISH, MAST-6 was found in first year sea ice in the Canadian Arctic Archipelago, with 20% of the cells containing at least one bacterium in their food vacuoles, suggesting that MAST-6, like MAST-1, are bacterivorous (Piwosz et al., 2013). In the Baltic Sea, MAST-6 cells were found to have both phytoplankton and bacteria in their food vacuoles suggesting that they are both algivorous and bacterivorous (Piwosz and Pernthaler, 2010). This clade of marine stramenopiles has been observed to prefer sediments across several coastal stations around Europe (Logares et al., 2012); however, our observations show that they are also important in pelagic systems in the Arctic.

Dinoflagellates had the highest relative abundance in August (24%), followed by April (14%) and June (10%); however, like with MAST, the dominant taxa varied by season. While both April and August were dominated by the *Gyrodinium* sp., *Gymnodinium* sp. was also abundant in August. While difficult to identify microscopically (Lovejoy, 2014; Kubiszyn and Wiktor, 2016), these two genera of naked heterotrophic dinoflagellates are abundant in 18S rRNA gene surveys of Arctic waters (Comeau et al., 2011; Marquardt et al., 2016). The dinoflagellate population in June was dominated by *Pelagodinium* sp. (7.6%), a member of the *Suessiaceae*. *Pelagodinium* is thought to be a symbiont of Foraminifera (Siano et al., 2010), but forams were a very small fraction of the protist communities, especially in June (<0.001%). The highest abundance of *Foraminifera* was observed in April (0.1%), but still was small compared to the relative abundance of *Pelagodinium* sp. in June. Given these observations, it is possible that we detected this symbiont during the free-living stage of its life cycle or that it is also a symbiont of other taxa abundant in June.

While typically less abundant than dinoflagellates in the Arctic, ciliates represent another important group of grazers in marine systems (e.g., Sherr et al., 1997). In line with microscopically obtained abundance estimates, dinoflagellate 18S rRNA gene sequences were always at least twice as abundant as ciliates regardless of season in a high-Arctic fjord (Marquardt et al., 2016). We observed more seasonality in the ratios of these two groups of protists, with ciliate sequences twice as abundant as dinoflagellate sequences in April and June, but less abundant in August (17%, compared to 24% dinoflagellates). This suggests that ciliates may play a more important role in coastal lagoon food webs than in other Arctic systems.

Oligotrich ciliates were the most abundant group of ciliates across all seasons, including *Strombidium* and *Laboea* (the latter only in August; 1.4%). OTUs classified as *Strombidium* were two times more abundant in June and August than April, while all months had a large percentage of reads that could not be classified beyond *Oligotrichia*, similar to other studies of ciliates in polar waters (Onda et al., 2017). *Strombidium* was found to be abundant in surface waters elsewhere in the Arctic, especially in the spring and summer, perhaps in part due to a mixotrophic lifestyle (Stoecker et al., 2017). *Strombidium* and *Laboea* have been observed to temporarily retain and gain energy from the chloroplasts from ingested diatoms or other phototrophic prey (Dolan and PÉrez, 2000). This could provide an energetic advantage over ciliates that rely solely on phagotrophy. April had higher relative abundances of ciliates belonging to the Mesodiniidae (3.1%) and Oligohymenophorea (specifically Scuticociliatia; 2.5%). Oligohymenophorea are strictly bacterivorous (Vaqué et al., 2008), but some Mesodiniidae species are mixotrophic, bordering autotrophic, with a preferred diet of cryptomonads as a source for harvested chloroplasts (McManus and Santoferrara, 2013). Cryptomonads were most abundant in April and August, in line with the distribution of Mesodiniidae OTUs. Mixotrophy was found to be the primary metabolism of ciliates in the oligotrophic waters of Fram Strait (Seuthe et al., 2011). The dominance of several potentially mixotrophic groups of ciliates in this study suggests that mixotrophy is also important in the coastal lagoons of the Beaufort Sea and could contribute to the overall productivity of these waters.

### Community Connectivity and Microbial Food Web

We used co-occurrence network analysis to investigate prokaryotic and eukaryotic community connectedness in each season using data from multiple years. We were unable to find similar seasonal networks for comparison in marine systems because most marine networks have been grouped by depth (Lima-Mendez et al., 2015; Milici et al., 2016) or were generated for entire time series datasets without seasonal breakdown (e.g., Chow et al., 2013). Still, our network sizes and clustering coefficients (Table 2) were within the range of these marine co-occurrence networks.

In one of the only aquatic microbial time series studies that performed seasonal network analysis, it was observed that network complexity of lake microbial communities was the greatest in spring, compared to summer or autumn (Kara et al., 2013). Similarly, we found that the June network was the largest, most connected, and most complex, having the highest clustering coefficient, highest connectance, and the lowest average path length, while the August network was the least complex and the complexity of under-ice network in April was intermediate. Kara et al. (2013) also noted that spring and autumn samples had the lowest and highest diversity, respectively, resulting in a negative relationship between network complexity and diversity. We did not observe this same relationship in the lagoons, possibly due to the contribution of freshwater bacteria and protists to the marine system and the formation of a diverse brackish microbial community in June. Given the same seasonal trends in network complexity between the lake study and our study, but differences in diversity-network complexity relationships, it is possible that seasonal trends in aquatic microbial network characteristics may be independent of the number of taxa present and driven more by ecosystem productivity, with food web complexity being highest during periods of high production.

River indicator taxa had the highest average node degree in June networks (Supplementary Figure S16) and common freshwater and brackish bacterial taxa accounted for the bulk of the significant correlations in the June networks (e.g., *Legionellales*, *Betaproteobacteria, Deltaproteobacteria*, and *Rhizobiales*, Figure 6). Whether these taxa were actively interacting or just happen to be passively co-existing is impossible to determine from this analysis, but it is important to note that river-impacted, nearshore systems may follow different diversity and connectivity patterns than lake or open ocean systems because mixing of freshwater and marine communities may elevate microbial diversity and form more complex microbial networks. The prevalence of freshwater and brackish microbial nodes in the spring network underscores the importance of these taxa in the coastal Arctic ecosystem during the spring freshest. Interestingly, two bacterial candidate phyla with small genomes, including WS3 and SR1 (Kantor et al., 2013; Farag et al., 2017), represented network “hubs” in June, with average node degrees >300. Microbes with streamlined genomes have also been observed to be network hubs in other studies of freshwater and marine systems (Peura et al., 2015; Milici et al., 2016), relying on interactions with other taxa in order to obtain metabolites that they cannot synthesize themselves.

Protists represented a small fraction of the nodes in the June networks, possibly due to the higher relative abundance of photoautotrophic protists during ice break-up. Most of the significant protistan relationships in June were between protists and bacteria rather than with other protists and were dominated by Chrysophytes (which can by mixotrophic; Beisser et al., 2017), diatoms, and heterotrophic groups of Rhizaria (Nakamura and Suzuki, 2015). These protists could be obtaining a necessary metabolite produced by co-located bacteria or, if heterotrophic or mixotrophic, could be grazing on bacteria, which can be enhanced during phytoplankton blooms (Hyun and Kim, 2003). Still it is important to note that the total number of significant protist-bacteria edges (11,256) in June exceeded those in April or August, but was far smaller than the number of Bac-Bac edges (94,983) and thus the relative contribution was less than in other months.

The April network was the next largest and contained the largest number (and percentage) of eukaryote nodes and Euk-Euk edges, especially among nodes belonging to the Syndiniales, other dinoflagellates, and ciliates (Figure 6A, Supplementary Figure S19). These relationships are in line with the known hosts for this group of parasites (Guillou et al., 2008; Torres-Beltrán et al., 2018) and support our hypothesis that parasitism was an important component of the under-ice food web. Extreme winter conditions in polar systems may increase parasitism due to environmental stress (e.g., low light, low *in situ* production). Syndiniales were also the most abundant group in co-occurrence networks generated as part of the Tara Oceans project from all regions sampled except for the Southern Ocean (the Arctic Ocean was not sampled as part of Tara Oceans; Lima-Mendez et al., 2015). As was observed at lower latitudes, these parasitic OTUs were most commonly correlated with other Syndiniales OTUs and with Dinophyceae OTUs. Syndiniales also correlated with several radiolarians, consistent with direct observations of similar associations through single-cell sequencing of radiolarians from a Norwegian fjord (Bråte et al., 2012), and with correlations between these protist groups under the ice north of Svalbard (Meshram et al., 2017). But unlike at lower latitudes (Lima-Mendez et al., 2015), correlations between Syndiniales and Ciliophora OTUs were common under the ice in the April. It is not possible to determine if this means that parasitism of ciliates is more prevalent in Arctic waters than at lower latitudes, but these observations suggest that correlations between putatively parasitic OTUs and presumed hosts under sea ice warrant continued investigation.

The August microbial community was less connected and more fragmented than other seasons, with a higher number of connected components, highest average path length (number of nodes needed to link individual nodes), and the lowest connectance (fraction of all possible links that are realized). A similar pattern was found for a Wisconsin lake in which the autumn network had the smallest network size and highest path length (Kara et al., 2013). The breakdown of microbial networks between June and August may be driven by physical processes such as increased mixing and reduced water column stratification, which was weaker in August than in June (Harris et al., 2017). Another possible explanation is that the August food web is not as reliant on fast energy transfer from large, fast-growing phytoplankton but rather on a slower energy transfer characteristic of a more detrital food web, the latter of which is often characterized by longer path lengths and generally weaker links (Rooney and McCann, 2012).

The August network featured the greatest percentage of negative relationships between nodes, suggesting more antagonistic relationships in the microbial food web in the summer than the spring (Figure 6). This is supported by our observation of high relative abundance of heterotrophic protist sequences in August. Furthermore, the concentration of phaeopigments was the highest in August (Connelly et al., 2015), which also supports high grazing on and microbial remineralization of phytoplankton-derived POM. If microbial populations were more focused on degrading or grazing on phytodetritus in August, this could result in fewer significant correlations among taxa and also longer chains within the food web as compared to other seasons. Bacterial networks associated with POM tended to be smaller than free-living networks in the Atlantic Ocean (Milici et al., 2016). Indeed common particle-associated bacteria (Flavobacteriales, Alteromonadales, and Rhodobacterales) made up a large component of our August networks (e.g., Buchan et al., 2014). Syndiniales, Dinophyceae, and ciliates were also abundant in the August network with a much more even distribution that in April, highlighting that both grazers and parasites were integral components of the August food web. The most connected protists belonged to the heterotrophic flagellate *Palpitomonas* (Yabuki et al., 2010) and the bacterivorous MAST-9, with a high percentage of significant negative correlations suggestive of an important predatory role.

## Conclusions

**Figure 7:**
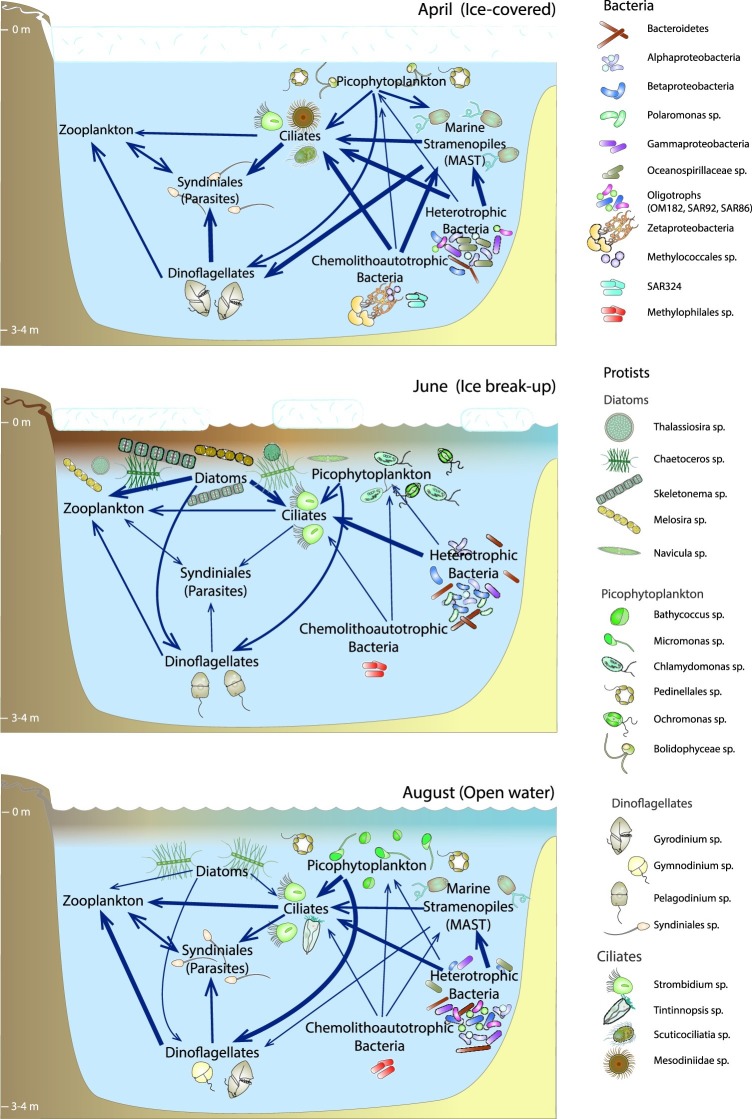
Diagram depicting major seasonal microbial community changes in the Beaufort Lagoons ecosystem, with arrows depicting hypothesized pathways for carbon flow from one group of organisms to another. The size of the arrow indicates relative magnitude of this hypothesized carbon flow, and the size of the text indicates relative size of the carbon pool, based on the relative abundance of microbial taxa each month. Many symbols after (Caron et al., 2017) or courtesy of the Integration and Application Network (ian.umces.edu/symbols/).

We observed strong seasonal changes in the composition and connectivity of bacterial and protistan communities in nearshore waters of the eastern Alaskan Beaufort Sea (Figures 2, 3, 7). Environmental conditions beneath sea ice favored parasitism and chemoautotrophy, including the surprising finding of Zetaproteobacteria. The presence of an increased relative abundance of chemoautotrophs suggests that iron, methane, nitrogen, and sulfur cycling are important under the ice during a time when the food web is often considered to be less productive. In the spring, we observed the formation of a complex and highly connected, brackish microbial community highlighting the importance and influence of terrestrial inputs into coastal marine ecosystems. Given the freshening of the Arctic Ocean, these microbes may become increasingly important in Arctic marine food webs in years to come. Nutrient depletion over the course of the summer favored a shift from a diatom-dominated food web to one characterized by an increased relative abundance of heterotrophic and mixotrophic protists, especially dinoflagellates, as well as picophototrophs *Micromonas* and *Bathycoccus* and other small phytoflagellates. Bacterial communities became increasingly enriched in common marine oligotrophic clades typically considered to have lower carbon demands and an increased ability to consume more recalcitrant organic matter. This shift to a more detrital food web in the late summer yielded a smaller and less connected network with longer paths between organisms than in April or June.

The Arctic is currently experiencing a number of physical changes that can have far-reaching effects on Arctic Ocean food webs. Surface air temperatures are warming at twice the rate of the rest of the globe, sea ice age and thickness continues to decline, and summer sea surface temperatures continue to show a warming trend year after year (Osborne et al., 2018). These changes are no doubt amplified in shallow, coastal systems such as our study system. These warmer temperatures also result in changes in precipitation and runoff patterns. We now have a baseline understanding of microbial communities in this region from which to predict community responses to a changing Arctic Ocean; one characterized by unique brackish communities with diatom blooms in the spring followed by long periods of nutrient-poor conditions in shallow waters inhabited by small grazers, picophototrophs, and oligotrophic bacterial clades. Continued long-term observations in this region are necessary to validate these predictions and assess their effects on higher trophic levels.

## Data Availability Statement

The datasets generated for this study can be found in the NCBI Short Read Archive BioProject # PRJNA530074 (SRR8832739-SRR8833063 for 16S rRNA and SRR8837972-SRR8838296 for 18S rRNA): https://www.ncbi.nlm.nih.gov/sra/PRJNA530074.

## Author Contributions

CK collected and processed samples, performed sequence and data analysis, and led writing. BC contributed to writing and interpretation. BC, JM, and KD conceptualized the project. JM and KD lead field efforts and collected samples. All authors contributed to manuscript revision, read, and approved the submitted version.

### Conflict of Interest

The authors declare that the research was conducted in the absence of any commercial or financial relationships that could be construed as a potential conflict of interest.

## Footnotes

^1^https://www.ncbi.nlm.nih.gov

^2^https://drive5.com/usearch/manual/uchime_algo.html

## References

[ref1] Alonso-Sáez L. Sánchez O. Gasol J. M. Balagué V. Pedrós-Alio C. (2008). Winter-to-summer changes in the composition and single-cell activity of near-surface Arctic prokaryotes. Environ. Microbiol. 10, 2444–2454. 10.1111/j.1462-2920.2008.01674.x 18557769

[ref2] Anderson M. J. (2017). “Permutational multivariate analysis of variance (PERMANOVA)” in Wiley stats ref: Statistics reference online eds. Balakrishnan N. Colton T. Everitt B. Piegorsch W. Ruggeri F. Teugels J. L. (Chichester, UK: John Wiley & Sons, Ltd), 1–15.

[ref3] Ardyna M. Babin M. Gosselin M. Devred E. Rainville L. Tremblay J.-É. (2014). Recent Arctic Ocean sea ice loss triggers novel fall phytoplankton blooms. Geophys. Res. Lett. 41, 6207–6212. 10.1002/2014GL061047

[ref4] Aronesty E. (2011). ea-utils: Command-line tools for processing biological sequencing data. Durham, NC. Available at: https://github.com/ExpressionAnalysis/ea-utils (Accessed March 01, 2016).

[ref5] Arrigo K. R. Perovich D. K. Pickart R. S. Brown Z. W. van Dijken G. L. Lowry K. E. (2012). Massive phytoplankton blooms under arctic sea ice. Science 336:1408. 10.1126/science.1215065 22678359

[ref6] Arrigo K. R. van Dijken G. L. (2015). Continued increases in Arctic Ocean primary production. Prog. Oceanogr. 136, 60–70. 10.1016/j.pocean.2015.05.002

[ref7] Azam F. Malfatti F. (2007). Microbial structuring of marine ecosystems. Nat. Rev. Microbiol. 5, 782–791. 10.1038/nrmicro1747 17853906

[ref8] Balzano S. Gourvil P. Siano R. Chanoine M. Marie D. Lessard S. (2012a). Diversity of cultured photosynthetic flagellates in the Northeast Pacific and Arctic oceans in summer. Biogeosciences 9, 4553–4571. 10.5194/bg-9-4553-2012

[ref9] Balzano S. Marie D. Gourvil P. Vaulot D. (2012b). Composition of the summer photosynthetic pico and nanoplankton communities in the Beaufort Sea assessed by T-RFLP and sequences of the 18S rRNA gene from flow cytometry sorted samples. ISME J. 6, 1480–1498. 10.1038/ismej.2011.213 22278671 PMC3400408

[ref309] Barker A. J. Douglas T. A. Jacobson A. D. McClelland J. W. Ilgen A. G. Khosh M. S. (2014). Late season mobilization of trace metals in two small Alaskan arctic watersheds as a proxy for landscape scale permafrost active layer dynamics. Chem. Geol. 381, 180–193. 10.1016/j.chemgeo.2014.05.012

[ref10] Beisser D. Graupner N. Bock C. Wodniok S. Grossmann L. Vos M. (2017). Comprehensive transcriptome analysis provides new insights into nutritional strategies and phylogenetic relationships of chrysophytes. PeerJ 5:e2832. 10.7717/peerj.2832 28097055 PMC5228505

[ref11] Bell L. Bluhm B. Iken K. (2016). Influence of terrestrial organic matter in marine food webs of the Beaufort Sea shelf and slope. Mar. Ecol. Prog. Ser. 550, 1–24. 10.3354/meps11725

[ref12] Benjamini Y. Hochberg Y. (1995). Controlling the false discovery rate: A practical and powerful approach to multiple. J. Roy. Stat. Soc. B Met. 57, 289–300.

[ref13] Booth B. C. Horner R. A. (1997). Microalgae on the Arctic Ocean section, 1994: species abundance and biomass. Deep Sea Res., Part II 44, 1607–1622. 10.1016/S0967-0645(97)00057-X

[ref14] Boyd E. S. Hamilton T. L. Havig J. R. Skidmore M. L. Shock E. L. (2014). Chemolithotrophic primary production in a subglacial ecosystem. Appl. Environ. Microbiol. 80, 6146–6153. 10.1128/AEM.01956-14, PMID: 25085483 PMC4178699

[ref15] Bråte J. Krabberød A. K. Dolven J. K. Ose R. F. Kristensen T. Bjørklund K. R. (2012). Radiolaria associated with large diversity of marine Alveolates. Protist 163, 767–777. 10.1016/J.PROTIS.2012.04.004 22658831

[ref16] Brown S. Bart J. Lanctot R. B. Johnson J. A. Kendall S. Payer D. (2007). Shorebird abundance and distribution of the coastal plain of the Arctic National Wildlife Refuge. Condor 109, 1–14.

[ref951] Buchan A. LeCleir G. R. Gulvik C. A. González J. M. (2014). Master recyclers: features and functions of bacteria associated with phytoplankton blooms. Nat. Rev. Microbiol. 12, 686–698. 10.1038/nrmicro3326 25134618

[ref17] Bunse C. Pinhassi J. (2017). Special series: microbial communities marine Bacterioplankton seasonal succession dynamics role of bacteria in marine biogeochemical cycling. Trends Microbiol. 25, 494–505. 10.1016/j.tim.2016.12.013 28108182

[ref18] Caporaso J. G. Kuczynski J. Stombaugh J. Bittinger K. Bushman F. D. Costello E. K.. (2010). QIIME allows analysis of high-throughput community sequencing data. Nat. Methods 7, 335–336. 10.1038/nmeth.f.303, PMID: 20383131 PMC3156573

[ref19] Caporaso J. G. Lauber C. L. Walters W. A. Berg-Lyons D. Lozupone C. A. Turnbaugh P. J. (2011). Global patterns of 16S rRNA diversity at a depth of millions of sequences per sample. Proc. Natl. Acad. Sci. USA 108, 4516–4522. 10.1073/pnas.1000080107 20534432 PMC3063599

[ref20] Carlsson P. Segatto A. Z. Graneli E. (1993). Nitrogen bound to humic matter of terrestrial origin-a nitrogen pool for coastal phytoplankton? Mar. Ecol. Prog. Ser. 97, 105–116. 10.3354/meps097105

[ref580] Caron D. A. Alexander H. Allen A. E. Archibald J. M. Armbrust E. V. Bachy C. (2017). Probing the evolution, ecology and physiology of marine protists using transcriptomics. Nat. Rev. Microbiol. 15, 6–20. 10.1038/nrmicro.2016.160 27867198

[ref21] Chao A. (1984). Nonparametric estimation of the number of classes in a population. Scand. J. Stat. 11, 265–270.

[ref22] Chow C.-E. T. Sachdeva R. Cram J. A. Steele J. A. Needham D. M. Patel A. (2013). Temporal variability and coherence of euphotic zone bacterial communities over a decade in the Southern California bight. ISME J. 7, 2259–2273. 10.1038/ismej.2013.122 23864126 PMC3834854

[ref23] Clarke K. R. (1993). Non-parametric multivariate analyses of changes in community structure. Austral Ecol. 18, 117–143. 10.1111/j.1442-9993.1993.tb00438.x

[ref24] Cleary A. C. Durbin E. G. (2016). Unexpected prevalence of parasite 18S rDNA sequences in winter among Antarctic marine protists. J. Plankton Res. 38, 401–417. 10.1093/plankt/fbw005

[ref301] Coffin R. B. Smith J. P. Plummer R. E. Yoza B. Larsen R. K. Millholland L. C. (2013). Spatial variation in shallow sediment methane sources and cycling on the Alaskan Beaufort Sea Shelf/Slope. Mar. Pet. Geol. 45, 186–197. 10.1016/j.marpetgeo.2013.05.002

[ref25] Comeau A. M. Li W. K. W. Tremblay J. É. Carmack E. C. Lovejoy C. (2011). Arctic Ocean microbial community structure before and after the 2007 record sea ice minimum. PLoS One 11:e27492. 10.1371/journal.pone.0027492, PMID: 22096583 PMC3212577

[ref260] Connelly T. L. Baer S. E. Cooper J. T. Bronk D. A. Wawrik B. (2014). Urea uptake and carbon fixation by marine pelagic bacteria and archaea during the Arctic summer and winter seasons. Appl. Environ. Microbiol. 80, 6013–6022. 10.1128/AEM.01431-14 25063662 PMC4178671

[ref26] Connelly T. McClelland J. Crump B. Kellogg C. Dunton K. (2015). Seasonal changes in quantity and composition of suspended particulate organic matter in lagoons of the Alaskan Beaufort Sea. Mar. Ecol. Prog. Ser. 527, 31–45. 10.3354/meps11207

[ref27] Corel E. Lopez P. Méheust R. Bapteste E. (2016). Network-thinking: graphs to analyze microbial complexity and evolution. Trends Microbiol. 24, 224–237. 10.1016/J.TIM.2015.12.003 26774999 PMC4766943

[ref28] Craig P. C. (1984). Fish use of coastal waters of the Alaska Beaufort Sea: a review. Trans. Am. Fish. Soc. 113, 265–282. 10.1577/1548-8659(1984)113<265:FUOCWO>2.0.CO;2

[ref29] Cram J. A. Chow C.-E. T. Sachdeva R. Needham D. M. Parada A. E. Steele J. A. (2015). Seasonal and interannual variability of the marine bacterioplankton community throughout the water column over ten years. ISME J. 9, 563–580. 10.1038/ismej.2014.153 25203836 PMC4331575

[ref30] de Vargas C. Audic S. Henry N. Decelle J. Mahé F. Logares R. (2015). Ocean plankton. Eukaryotic plankton diversity in the sunlit ocean. Science 348:1261605. 10.1126/science.1261605 25999516

[ref31] Dolan J. R. PÉrez M. T. (2000). Costs, benefits and characteristics of mixotrophy in marine oligotrichs. Freshw. Biol. 45, 227–238. 10.1046/j.1365-2427.2000.00659.x

[ref32] Dufrêne M. Legendre P. (1997). Species assemblages and indicator species: the need for a flexible asymmetrical approach. Ecol. Monogr. 67, 345–366. 10.1890/0012-9615(1997)067[0345,SAAIST]2.0.CO;2

[ref33] Dunton K. H. Schonberg S. V. Cooper L. W. (2012). Food web structure of the Alaskan Nearshore shelf and estuarine lagoons of the Beaufort Sea. Estuar. Coasts 35, 416–435. 10.1007/s12237-012-9475-1

[ref34] Dunton K. H. Weingartner T. Carmack E. C. (2006). The nearshore western Beaufort Sea ecosystem: circulation and importance of terrestrial carbon in arctic coastal food webs. Prog. Oceanogr. 71, 362–378. 10.1016/j.pocean.2006.09.011

[ref35] Edgar R. C. (2013). UPARSE: highly accurate OTU sequences from microbial amplicon reads. Nat. Methods 10, 996–998. 10.1038/nmeth.2604, PMID: 23955772

[ref36] Faith D. P. (1992). Conservation evaluation and phylogentic diversity. Biol. Conserv. 61, 1–10.

[ref37] Falkowski P. G. Fenchel T. Delong E. F. (2008). The microbial engines that drive Earth’s biogeochemical cycles. Science 320, 1034–1039. 10.1126/science.1153213, PMID: 18497287

[ref38] Farag I. F. Youssef N. H. Elshahed M. S. (2017). Global distribution patterns and Pangenomic diversity of the candidate phylum “Latescibacteria” (WS3). Appl. Environ. Microbiol. 83, e00521–e00517. 10.1128/AEM.00521-17 28314726 PMC5411500

[ref39] Faust K. Sathirapongsasuti J. F. Izard J. Segata N. Gevers D. Raes J.. (2012). Microbial co-occurrence relationships in the human microbiome. PLoS Comput. Biol. 8:e1002606. 10.1371/journal.pcbi.1002606, PMID: 22807668 PMC3395616

[ref40] Fernández-Gómez B. Díez B. Polz M. F. Arroyo J. I. Alfaro F. D. Marchandon G. (2019). Bacterial community structure in a sympagic habitat expanding with global warming: brackish ice brine at 85–90 °N. ISME J. 13, 316–333. 10.1038/s41396-018-0268-9 30228379 PMC6331608

[ref41] Ferrera I. Sebastian M. Acinas S. G. Gasol J. M. (2015). Prokaryotic functional gene diversity in the sunlit ocean: stumbling in the dark. Curr. Opin. Microbiol. 25, 33–39. 10.1016/j.mib.2015.03.007 25863027

[ref42] Fuhrman J. A. Cram J. A. Needham D. M. (2015). Marine microbial community dynamics and their ecological interpretation. Nat. Rev. Microbiol. 13, 133–146. 10.1038/nrmicro3417, PMID: 25659323

[ref43] Garneau M.-È. Roy S. Lovejoy C. Gratton Y. Vincent W. F. (2008). Seasonal dynamics of bacterial biomass and production in a coastal arctic ecosystem: Franklin Bay, western Canadian Arctic. J. Geophys. Res. 113:C07S91. 10.1029/2007JC004281

[ref44] Ghiglione J.-F. Galand P. E. Pommier T. Pedros-Alio C. Maas E. W. Bakker K. (2012). Pole-to-pole biogeography of surface and deep marine bacterial communities. Proc. Natl. Acad. Sci. USA 109, 17633–17638. 10.1073/pnas.1208160109 23045668 PMC3491513

[ref45] Gilbert J. A. Steele J. A. Caporaso J. G. Steinbrück L. Reeder J. Temperton B.. (2012). Defining seasonal marine microbial community dynamics. ISME J. 6, 298–308. 10.1038/ismej.2011.107, PMID: 21850055 PMC3260500

[ref46] Gu Z. Gu L. Eils R. Schlesner M. Brors B. (2014). Circlize implements and enhances circular visualization in R. Bioinformatics 30, 2811–2812. 10.1093/bioinformatics/btu393 24930139

[ref47] Guidi L. Chaffron S. Bittner L. Eveillard D. Larhlimi A. Roux S.. (2016). Plankton networks driving carbon export in the oligotrophic ocean. Nature 532, 465–470. 10.1038/nature16942, PMID: 26863193 PMC4851848

[ref48] Guillou L. Viprey M. Chambouvet A. Welsh R. M. Kirkham A. R. Massana R. (2008). Widespread occurrence and genetic diversity of marine parasitoids belonging to Syndiniales (Alveolata). 10, 3349–3365. 10.1111/j.1462-2920.2008.01731.x 18771501

[ref49] Hardge K. Peeken I. Neuhaus S. Lange B. A. Stock A. Stoeck T. (2017). The importance of sea ice for exchange of habitat-specific protist communities in the Central Arctic Ocean. J. Mar. Syst. 165, 124–138. 10.1016/j.jmarsys.2016.10.004

[ref50] Harris C. M. McClelland J. W. Connelly T. L. Crump B. C. Dunton K. H. (2017). Salinity and temperature regimes in eastern Alaskan Beaufort Sea lagoons in relation to source water contributions. Estuar. Coasts 40, 50–62. 10.1007/s12237-016-0123-z

[ref51] Harris C. M. McTigue N. D. McClelland J. W. Dunton K. H. (2018). Do high Arctic coastal food webs rely on a terrestrial carbon subsidy? Food Webs 15:e00081. 10.1016/j.fooweb.2018.e00081

[ref52] Hernández-Ruiz M. Prieto A. Barber-Lluch E. Teira E. (2018). Amino acid utilization by eukaryotic picophytoplankton in a coastal upwelling system. Mar. Ecol. Prog. Ser. 588, 43–57. 10.3354/meps12435

[ref53] Holmes R. M. McClelland J. W. Peterson B. J. Tank S. E. Bulygina E. Eglinton T. I. (2012). Seasonal and annual fluxes of nutrients and organic matter from large Rivers to the Arctic Ocean and surrounding seas. Estuar. Coasts 35, 369–382. 10.1007/s12237-011-9386-6

[ref54] Hugerth L. W. Larsson J. Alneberg J. Lindh M. V. Legrand C. Pinhassi J. (2015). Metagenome-assembled genomes uncover a global brackish microbiome. Genome Biol. 16:279. 10.1186/s13059-015-0834-7 26667648 PMC4699468

[ref55] Hyun J. Kim K. (2003). Bacterial abundance and production during the unique spring phytoplankton bloom in the Central Yellow Sea. Mar. Ecol. Prog. Ser. 252, 77–88. 10.3354/meps252077

[ref56] Johnson J. A. Lanctot R. B. Andres B. A. Bart J. R. Brown S. C. Kendall S. J. (2007). Predicting breeding shorebird distributions on the Arctic coastal plain of Alaska. Arctic 60, 277–293. 10.1890/ES12-00292.1

[ref57] Joli N. Monier A. Logares R. Lovejoy C. (2017). Seasonal patterns in Arctic prasinophytes and inferred ecology of *Bathycoccus* unveiled in an Arctic winter metagenome. ISME J. 117, 1372–1385. 10.1038/ismej.2017.7. PMC5437359 28267153

[ref58] Kantor R. S. Wrighton K. C. Handley K. M. Sharon I. Hug L. A. Castelle C. J. (2013). Small genomes and sparse metabolisms of sediment-associated bacteria from four candidate phyla. MBio 4, e00708–e00713. 10.1128/mBio.00708-13 24149512 PMC3812714

[ref59] Kara E. L. Hanson P. C. Hu Y. H. Winslow L. McMahon K. D. (2013). A decade of seasonal dynamics and co-occurrences within freshwater bacterioplankton communities from eutrophic Lake Mendota, WI, USA. ISME J. 7, 680–684. 10.1038/ismej.2012.118, PMID: 23051691 PMC3578560

[ref60] Kilias E. S. Peeken I. Metfies K. (2014). Insight into protist diversity in Arctic Sea ice and melt-pond aggregate obtained by pyrosequencing view supplementary material. Polar Res. 33, 23466. 10.3402/polar.v33.23466

[ref61] Kubiszyn A. M. Wiktor J. M. (2016). The *Gymnodinium* and *Gyrodinium* (Dinoflagellata: Gymnodiniaceae) of the West Spitsbergen waters (1999–2010): biodiversity and morphological description of unidentified species. Polar Biol. 39, 1739–1747. 10.1007/s00300-015-1764-2

[ref304] Kvenvolden K. V. Lilley M. D. Lorenson T. D. Barnes P. W. McLaughlin E. (1993). The Beaufort Sea continental shelf as a seasonal source of atmospheric methane. Geophys. Res. Lett. 20, 2459–2462. 10.1029/93GL02727

[ref62] Li W. K. W. McLaughlin F. A. Lovejoy C. Carmack E. C. (2009). Smallest algae thrive as the Arctic Ocean freshens. Science 326:539. 10.1126/science.1179798, PMID: 19900890

[ref63] Lima-Mendez G. Faust K. Henry N. Decelle J. Colin S. Carcillo F. (2015). Determinants of community structure in the global plankton interactome. Science 348:1262073. 10.1126/science.1262073 25999517

[ref64] Logares R. Audic S. Santini S. Pernice M. C. de Vargas C. Massana R. (2012). Diversity patterns and activity of uncultured marine heterotrophic flagellates unveiled with pyrosequencing. ISME J. 6, 1823–1833. 10.1038/ismej.2012.36, PMID: 22534609 PMC3446805

[ref65] Lovejoy C. (2014). Changing views of Arctic Protists (marine microbial eukaryotes) in a changing Arctic. Acta Protozool. 53, 91–100. 10.4467/16890027AP.14.009.1446

[ref66] Lovejoy C. Galand P. E. Kirchman D. L. (2011). Picoplankton diversity in the Arctic Ocean and surrounding seas. Mar. Biodivers. 41, 5–12. 10.1007/s12526-010-0062-z

[ref67] Lovejoy C. Massana R. Pedrós-Alió C. (2006). Diversity and distribution of marine microbial eukaryotes in the Arctic Ocean and adjacent seas. Appl. Environ. Microbiol. 72, 3085–3095. 10.1128/AEM.72.5.3085-3095.2006, PMID: 16672445 PMC1472370

[ref302] Lorenson T. D. Grienert J. Coffin R. B. (2016). Dissolved methane in the Beaufort Sea and the Arctic Ocean, 1992–2009; sources and atmospheric flux. Limnol. Oceanogr. 61, S300–S323. 10.1002/lno.10457

[ref68] Marchese C. Albouy C. Tremblay J.-É. Dumont D. D’Ortenzio F. Vissault S. (2017). Changes in phytoplankton bloom phenology over the north water (NOW) polynya: a response to changing environmental conditions. Polar Biol. 40, 1721–1737. 10.1007/s00300-017-2095-2

[ref69] Marquardt M. Vader A. Stübner E. I. Reigstad M. Gabrielsen T. M. (2016). Strong seasonality of marine microbial eukaryotes in a high-Arctic Fjord (Isfjorden, in West Spitsbergen, Norway). Appl. Environ. Microbiol. 82, 1868–1880. 10.1128/AEM.03208-15 26746718 PMC4784050

[ref70] McAllister S. M. Davis R. E. McBeth J. M. Tebo B. M. Emerson D. Moyer C. L. (2011). Biodiversity and emerging biogeography of the neutrophilic iron-oxidizing Zetaproteobacteria. Appl. Environ. Microbiol. 77, 5445–5457. 10.1128/AEM.00533-11 21666021 PMC3147450

[ref71] McBeth J. M. Little B. J. Ray R. I. Farrar K. M. Emerson D. (2011). Neutrophilic iron-oxidizing “Zetaproteobacteria” and mild steel corrosion in nearshore marine environments. Appl. Environ. Microbiol. 77, 1405–1412. 10.1128/AEM.02095-10 21131509 PMC3067224

[ref72] McCallister S. L. Bauer J. E. Cherrier J. E. Ducklow H. W. (2004). Assessing sources and ages of organic matter supporting river and estuarine bacterial production: a multiple isotope (D14C, d13C, and d15N) approach. Limnol. Oceanogr. 49, 1687–1702. 10.4319/lo.2004.49.5.1687

[ref73] McClelland J. W. Déry S. J. Peterson B. J. Holmes R. M. Wood E. F. (2006). A pan-arctic evaluation of changes in river discharge during the latter half of the 20th century. Geophys. Res. Lett. 33:L06715. 10.1029/2006GL025753

[ref74] McClelland J. W. Townsend-Small A. Holmes R. M. Pan F. Stieglitz M. Khosh M. (2014). River export of nutrients and organic matter from the north slope of Alaska to the Beaufort Sea. Water Resour. Res. 50, 1823–1839. 10.1002/2013WR014722

[ref75] McManus G. B. Santoferrara L. F. (2013). “Tintinnids in microzooplankton communities” in The biology and ecology of Tintinnid ciliates. eds. Dolan J. R. Montagnes D. J. S. Agatha S. Coats D. W. Stoecker D. K. (Chichester, UK: John Wiley & Sons, Ltd), 198–213.

[ref76] McMurdie P. J. Holmes S. (2014). Waste Not, want Not: why rarefying microbiome data is inadmissible. PLoS Comput. Biol. 10:e1003531. 10.1371/journal.pcbi.1003531, PMID: 24699258 PMC3974642

[ref77] McPhee M. G. Proshutinsky A. Morison J. H. Steele M. Alkire M. B. (2009). Rapid change in freshwater content of the Arctic Ocean. Geophys. Res. Lett. 36:L10602. 10.1029/2009GL037525

[ref78] Meshram A. R. Vader A. Kristiansen S. Gabrielsen T. M. (2017). Microbial eukaryotes in an Arctic under-ice Spring bloom north of Svalbard. Front. Microbiol. 8:1099. 10.3389/fmicb.2017.01099 28702000 PMC5487457

[ref79] Meyer K. A. O’Neil J. M. Hitchcock G. L. (2014). Microbial production along the West Florida shelf: responses of bacteria and viruses to the presence and phase of Karenia brevis blooms. Harmful Algae 38, 110–118. 10.1016/j.hal.2014.04.015

[ref80] Michel C. Legendre L. Therriault J. C. Dcmers S. Vandevelde T. (1993). Springtime coupling between ice algal and phytoplankton assemblages in southeastern Hudson Bay, Canadian Arctic. Polar Biol. 13, 441–449. 10.1007/BF00233135

[ref81] Milici M. Deng Z.-L. Tomasch J. Decelle J. Wos-Oxley M. L. Wang H.. (2016). Co-occurrence analysis of microbial taxa in the Atlantic Ocean reveals high connectivity in the free-living bacterioplankton. Front. Microbiol. 7:649. 10.3389/fmicb.2016.00649, PMID: 27199970 PMC4858663

[ref82] Mohan S. D. Connelly T. L. Harris C. M. Dunton K. H. Mcclelland J. W. (2016). Seasonal trophic linkages in Arctic marine invertebrates assessed *via* fatty acids and compound-specific stable isotopes. Ecosphere 7:e01429. 10.1002/ecs2.1429

[ref83] Monier A. Comte J. Babin M. Forest A. Matsuoka A. Lovejoy C. (2015). Oceanographic structure drives the assembly processes of microbial eukaryotic communities. ISME J. 9, 990–1002. 10.1038/ismej.2014.197, PMID: 25325383 PMC4817713

[ref84] Monier A. Terrado R. Thaler M. Comeau A. Medrinal E. Lovejoy C. (2013). Upper Arctic Ocean water masses harbor distinct communities of heterotrophic flagellates. Biogeosciences 10, 4273–4286. 10.5194/bg-10-4273-2013

[ref85] Morata N. Renaud P. Brugel S. Hobson K. Johnson B. (2008). Spatial and seasonal variations in the pelagic–benthic coupling of the southeastern Beaufort Sea revealed by sedimentary biomarkers. Mar. Ecol. Prog. Ser. 371, 47–63. 10.3354/meps07677

[ref86] Morison J. Kwok R. Peralta-Ferriz C. Alkire M. Rigor I. Andersen R. (2012). Changing Arctic Ocean freshwater pathways. Nature 481, 66–70. 10.1038/nature10705 22222749

[ref87] Nakamura Y. Suzuki N. (2015). “Phaeodaria: diverse marine cercozoans of world-wide distribution” in Marine Protists. eds. Ohtsuka S. Suzaki T. Horiguchi T. Suzuki N. Not F. (Tokyo: Springer Japan), 223–249.

[ref88] Nolan M. Churchwell R. Adams J. McClelland J. Tape K. D. Kendall S. (2011). “Predicting the impact of glacier loss on fish, birds, floodplains, and estuaries in the Arctic National Wildlife Refuge” in Observing, studying and managing for change. Proceedings of the fourth interagency conference on research in the watersheds: U.S. Geological Survey scientific investigations report. eds. Medley C. N. Patterson G. Parker M. J. (Reston, VA: U.S. Geological Survey), 49–54.

[ref89] Oksanen J. Blanchet F. G. Friendly M. Kindt R. Legendre P. McGlinn D. (2019). vegan: Community Ecology Package. *R Packag. version 2.5-4*. Available at: https://cran.r-project.org/package=vegan (Accessed February 04, 2019).

[ref90] Onda D. F. L. Medrinal E. Comeau A. M. Thaler M. Babin M. Lovejoy C. (2017). Seasonal and interannual changes in ciliate and *Dinoflagellate* species assemblages in the Arctic Ocean (Amundsen gulf, Beaufort Sea, Canada). Front. Mar. Sci. 4:16. 10.3389/fmars.2017.00016

[ref91] Osborne E. Richter-Menge J. Jeffries M. (2018). Arctic Report Card 2018. Available at: https://www.arctic.noaa.gov/Report-Card (Accessed April 08, 2019).

[ref92] Peres-Neto P. R. Jackson D. A. (2001). How well do multivariate data sets match? The advantages of a procrustean superimposition approach over the mantel test. Oecologia 129, 169–178. 10.1007/s004420100720 28547594

[ref93] Peura S. Bertilsson S. Jones R. I. Eiler A. (2015). Resistant microbial Cooccurrence patterns inferred by network topology. Appl. Environ. Microbiol. 81, 2090–2097. 10.1128/AEM.03660-14, PMID: 25576616 PMC4345367

[ref94] Piwosz K. Pernthaler J. (2010). Seasonal population dynamics and trophic role of planktonic nanoflagellates in coastal surface waters of the southern Baltic Sea. Environ. Microbiol. 12, 364–377. 10.1111/j.1462-2920.2009.02074.x 19799618

[ref95] Piwosz K. Wiktor J. M. Niemi A. Tatarek A. Michel C. (2013). Mesoscale distribution and functional diversity of picoeukaryotes in the first-year sea ice of the Canadian Arctic. ISME J. 7, 1461–1471. 10.1038/ismej.2013.39, PMID: 23514779 PMC3721110

[ref975] Pokrovsky O. S. Manasypov R. M. Loiko S. V. Krickov I. A. Kopysov S. G. Kolesnichenko L. G. (2016). Trace element transport in western Siberian rivers across a permafrost gradient. Biogeosciences 13, 1877–1900. 10.5194/bg-13-1877-2016

[ref96] Poulin M. Daugbjerg N. Gradinger R. Ilyash L. Ratkova T. von Quillfeldt C. (2011). The pan-Arctic biodiversity of marine pelagic and sea-ice unicellular eukaryotes: a first-attempt assessment. Mar. Biodivers. 41, 13–28. 10.1007/s12526-010-0058-8

[ref300] Quaiser A. Bodi X. Dufresne A. Naquin D. Francez A. J. Dheilly A. (2014). Unraveling the stratification of an iron-oxidizing microbial mat by metatranscriptomics. PLoS One 9. 10.1371/journal.pone.0102561 PMC4102501 25033299

[ref97] Quast C. Pruesse E. Yilmaz P. Gerken J. Schweer T. Yarza P.. (2013). The SILVA ribosomal RNA gene database project: improved data processing and web-based tools. Nucleic Acids Res. 41, D590–D596. 10.1093/nar/gks1219, PMID: 23193283 PMC3531112

[ref940] Rember R. D. Trefry J. H. (2004). Increased concentrations of dissolved trace metals and organic carbon during snowmelt in rivers of the alaskan arctic. Geochim. Cosmochim. Acta 68, 477–489. 10.1016/S0016-7037(03)00458-7

[ref303] Riedel M. Brent T. A. Taylor G. Taylor A. E. Hong J. K. Jin Y. K. (2014). Evidence for gas hydrate occurrences in the Canadian Arctic Beaufort Sea within permafrost-associated shelf and deep-water marine environments. Mar. Pet. Geol. 81, 66–78. 10.1016/j.marpetgeo.2016.12.027

[ref98] Rooney N. McCann K. S. (2012). Integrating food web diversity, structure and stability. Trends Ecol. Evol. 27, 40–46. 10.1016/J.TREE.2011.09.001 21944861

[ref99] Satomi M. Fujii T. (2014). “The family oceanospirillaceae” in The prokaryotes. eds. Rosenberg E. DeLong E. F. Lory S. Stackebrandt E. Thompson F. (Berlin, Heidelberg: Springer Berlin Heidelberg), 491–527.

[ref100] Schreiner K. M. Bianchi T. S. Eglinton T. I. Allison M. A. Hanna A. J. M. (2013). Sources of terrigenous inputs to surface sediments of the Colville River Delta and Simpson’s lagoon, Beaufort Sea, Alaska. J. Geophys. Res. Biogeosci. 118, 808–824. 10.1002/jgrg.20065

[ref101] Serreze M. C. Barry R. G. (2011). Processes and impacts of Arctic amplification: a research synthesis. Glob. Planet. Chang. 77, 85–96. 10.1016/j.gloplacha.2011.03.004

[ref102] Seuthe L. Töpper B. Reigstad M. Thyrhaug R. Vaquer-Sunyer R. (2011). Microbial communities and processes in ice-covered Arctic waters of the northwestern Fram Strait (75 to 80°N) during the vernal pre-bloom phase. Aquat. Microb. Ecol. 64, 253–266. 10.3354/ame01525

[ref970] Shakhova N. Semiletov I. Gustafsson O. Sergienko V. Lobkovsky L. Dudarev O. (2017). Current rates and mechanisms of subsea permafrost degradation in the East Siberian Arctic Shelf. Nat. Commun. 8:15872. 10.1038/ncomms15872 28639616 PMC5489687

[ref103] Sheik C. S. Jain S. Dick G. J. (2014). Metabolic flexibility of enigmatic SAR324 revealed through metagenomics and metatranscriptomics. Environ. Microbiol. 16, 304–317. 10.1111/1462-2920.12165 23809230

[ref104] Sherr E. B. Sherr B. F. Fessenden L. (1997). Heterotrophic protists in the Central Arctic Ocean. Deep Sea Res., Part II 44, 1665–1682. 10.1016/S0967-0645(97)00050-7

[ref105] Siano R. Montresor M. Probert I. Not F. de Vargas C. (2010). Pelagodinium gen. Nov. and P. béii comb. nov., a Dinoflagellate Symbiont of planktonic foraminifera. Protist 161, 385–399. 10.1016/j.protis.2010.01.002, PMID: 20149979

[ref971] Singer E. Emerson D. Webb E. A. Barco R. A. Kuenen J. G. Nelson W. C. (2011). Mariprofundus ferrooxydans PV-1 the first genome of a marine Fe(II) oxidizing Zetaproteobacterium. PLoS One 6. 10.1371/journal.pone.0025386 PMC3179512 21966516

[ref107] Sipler R. E. Kellogg C. T. E. Connelly T. L. Roberts Q. N. Yager P. L. Bronk D. A. (2017). Microbial community response to terrestrially derived dissolved organic matter in the coastal Arctic. Front. Microbiol. 8:1018. 10.3389/fmicb.2017.01018 28649233 PMC5465303

[ref108] Smith M. W. Herfort L. Fortunato C. S. Crump B. C. Simon H. M. (2017). Microbial players and processes involved in phytoplankton bloom utilization in the water column of a fast-flowing, river-dominated estuary. Microbiology 6:e00467. 10.1002/mbo3.467 PMC5552926 28318115

[ref109] Smith B. Wilson J. B. (1996). A consumer’s guide to evenness indices. Oikos 76, 70–82. 10.2307/3545749

[ref110] Smoot M. E. Ono K. Ruscheinski J. Wang P.-L. Ideker T. (2011). Cytoscape 2.8: new features for data integration and network visualization. Bioinformatics 27, 431–432. 10.1093/bioinformatics/btq675, PMID: 21149340 PMC3031041

[ref111] Spring S. Scheuner C. Göker M. Klenk H.-P. (2015). A taxonomic framework for emerging groups of ecologically important marine gammaproteobacteria based on the reconstruction of evolutionary relationships using genome-scale data. Front. Microbiol. 6:281. 10.3389/fmicb.2015.00281, PMID: 25914684 PMC4391266

[ref113] Stoecker D. K. Hansen P. J. Caron D. A. Mitra A. (2017). Mixotrophy in the marine plankton. Annu. Rev. Mar. Sci. 9, 311–335. 10.1146/annurev-marine-010816-060617, PMID: 27483121

[ref114] Stroeve J. C. Serreze M. C. Holland M. M. Kay J. E. Malanik J. Barrett A. P. (2012). The Arctic’s rapidly shrinking sea ice cover: a research synthesis. Clim. Chang. 110, 1005–1027. 10.1007/s10584-011-0101-1

[ref115] Taylor A. R. Lanctot R. B. Powell A. N. Huettman F. Nigro D. A. Kendall S. J. (2010). Distribution and community characteristics of staging shorebirds on the northern coast of Alaska. Arctic 63, 451–467. 10.14430/arctic3334

[ref116] Teeling H. Fuchs B. M. Becher D. Klockow C. Gardebrecht A. Bennke C. M. (2012). Substrate-controlled succession of marine Bacterioplankton populations induced by a phytoplankton bloom. Science 336, 608–611. 10.1126/science.1218344 22556258

[ref117] Terrado R. Scarcella K. Thaler M. Vincent W. F. Lovejoy C. (2013). Small phytoplankton in Arctic seas: vulnerability to climate change. Biodiversity 14, 2–18. 10.1080/14888386.2012.704839

[ref118] Terrado R. Vincent W. Lovejoy C. (2009). Mesopelagic protists: diversity and succession in a coastal Arctic ecosystem. Aquat. Microb. Ecol. 56, 25–39. 10.3354/ame01327

[ref119] Thaler M. Lovejoy C. (2013). Environmental selection of marine stramenopile clades in the Arctic Ocean and coastal waters. Polar Biol. 37, 347–357. 10.1007/s00300-013-1435-0

[ref120] Thaler M. Lovejoy C. (2015). Biogeography of heterotrophic flagellate populations indicates the presence of generalist and specialist taxa in the Arctic Ocean. Appl. Environ. Microbiol. 81, 2137–2148. 10.1128/AEM.02737-14, PMID: 25595764 PMC4345384

[ref121] Timmermans M.-L. Ladd C. (2018). Sea Surface Temperature. Available at: https://www.arctic.noaa.gov/Report-Card (Accessed March 21, 2019).

[ref122] Torres-Beltrán M. Sehein T. Pachiadaki M. G. Hallam S. J. Edgcomb V. (2018). Protistan parasites along oxygen gradients in a seasonally anoxic fjord: a network approach to assessing potential host-parasite interactions. Deep Sea Res., Part II. 156, 97–110. 10.1016/j.dsr2.2017.12.026

[ref123] Vaqué D. Guadayol Ò. Peters F. Felipe J. Angel-Ripoll L. Terrado R. (2008). Seasonal changes in planktonic bacterivory rates under the ice-covered coastal Arctic Ocean. Limnol. Oceanogr. 53, 2427–2438. 10.4319/lo.2008.53.6.2427

[ref124] Veillette J. Lovejoy C. Potvin M. Harding T. Jungblut A. D. Antoniades D. (2011). Milne fiord epishelf lake: a coastal Arctic ecosystem vulnerable to climate change. Écoscience 18, 304–316. 10.2980/18-3-3443

[ref125] Vernet M. Richardson T. L. Metfies K. Nöthig E.-M. Peeken I. (2017). Models of plankton community changes during a warm water anomaly in Arctic waters show altered trophic pathways with minimal changes in carbon export. Front. Mar. Sci. 4:160. 10.3389/fmars.2017.00160

[ref126] Vick-Majors T. J. Mitchell A. C. Achberger A. M. Christner B. C. Dore J. E. Michaud A. B.. (2016). Physiological ecology of microorganisms in subglacial Lake Whillans. Front. Microbiol. 7:1705. 10.3389/fmicb.2016.01705, PMID: 27833599 PMC5081474

[ref127] von Biela V. R. Zimmerman C. E. Cohn B. R. Welker J. M. (2013). Terrestrial and marine trophic pathways support young-of-year growth in a nearshore Arctic fish. Polar Biol. 36, 137–146. 10.1007/s00300-012-1244-x

[ref128] Wassmann P. Duarte C. M. Agustí S. Sejr M. K. (2011). Footprints of climate change in the Arctic marine ecosystem. Glob. Chang. Biol. 17, 1235–1249. 10.1111/j.1365-2486.2010.02311.x

[ref129] Weiss S. Van Treuren W. Lozupone C. Faust K. Friedman J. Deng Y. (2016). Correlation detection strategies in microbial data sets vary widely in sensitivity and precision. ISME J. 10, 1669–1681. 10.1038/ismej.2015.235 26905627 PMC4918442

[ref131] Whitefield J. Winsor P. McClelland J. Menemenlis D. (2015). A new river discharge and river temperature climatology data set for the pan-Arctic region. Ocean Model 88, 1–15. 10.1016/j.ocemod.2014.12.012

[ref132] Williams R. J. Berlow E. L. Dunne J. A. Barabá Si A.-L. Martinez N. D. (2002). Two degrees of separation in complex food webs. P. Natl. Acad. Sci. USA. 99, 12913–12916. 10.1073/pnas.192448799 PMC130559 12235367

[ref133] Worden A. Z. Follows M. J. Giovannoni S. J. Wilken S. Zimmerman A. E. Keeling P. J. (2015). Rethinking the marine carbon cycle: factoring in the multifarious lifestyles of microbes. Science 347, 1257594–1257594. 10.1126/science.1257594 25678667

[ref134] Yabuki A. Inagaki Y. Ishida K. (2010). Palpitomonas bilix gen. Et sp. nov.: a novel deep-branching heterotroph possibly related to Archaeplastida or Hacrobia. Protist 161, 523–538. 10.1016/J.PROTIS.2010.03.001 20418156

[ref135] Yilmaz P. Parfrey L. W. Yarza P. Gerken J. Pruesse E. Quast C. (2014). The SILVA and “all-species living tree project (LTP)” taxonomic frameworks. Nucleic Acids Res. 42, D643–D648. 10.1093/nar/gkt1209 24293649 PMC3965112

[ref136] Yunker M. B. Backus S. M. Graf Pannatier E. Jeffries D. S. Macdonald R. W. (2002). Sources and significance of alkane and PAH hydrocarbons in Canadian Arctic rivers. Estuar. Coast. Shelf Sci. 55, 1–31. 10.1006/ecss.2001.0880

[ref137] Yunker M. B. Macdonald R. W. Cretney W. J. Fowler B. R. McLaughlin F. A. (1993). Alkane, terpene and polycyclic aromatic hydrocarbon geochemistry of the Mackenzie River and Mackenzie shelf: riverine contributions to Beaufort Sea coastal sediment. Geochim. Cosmochim. Acta 57, 3041–3061. 10.1016/0016-7037(93)90292-5

