# Supplementary material for: Strong Seasonality in Arctic Estuarine Microbial Food Webs
Source: Front Microbiol. 2019 Nov 29;10:2628. doi: 10.3389/fmicb.2019.02628 (PMC6896822; doi:10.3389/fmicb.2019.02628)
Supplement: Supplementary file 1 [file Data_Sheet_1.pdf]

Strong seasonality in arctic estuarine microbial food webs

Supplementary Material

doi: 10.3389/fmicb.2019.02628

Colleen T. E. Kellogg[1], James W. McClelland[2], Kenneth H. Dunton[2], Byron C. Crump[3]

[1] Hakai Institute, Heriot Bay, BC, Canada,
[2] The University of Texas at Austin Marine Science Institute, Port Aransas, TX 78373, USA
[3] College of Earth, Ocean, and Atmospheric Sciences, Oregon State University, Corvallis, OR, 97331, USA

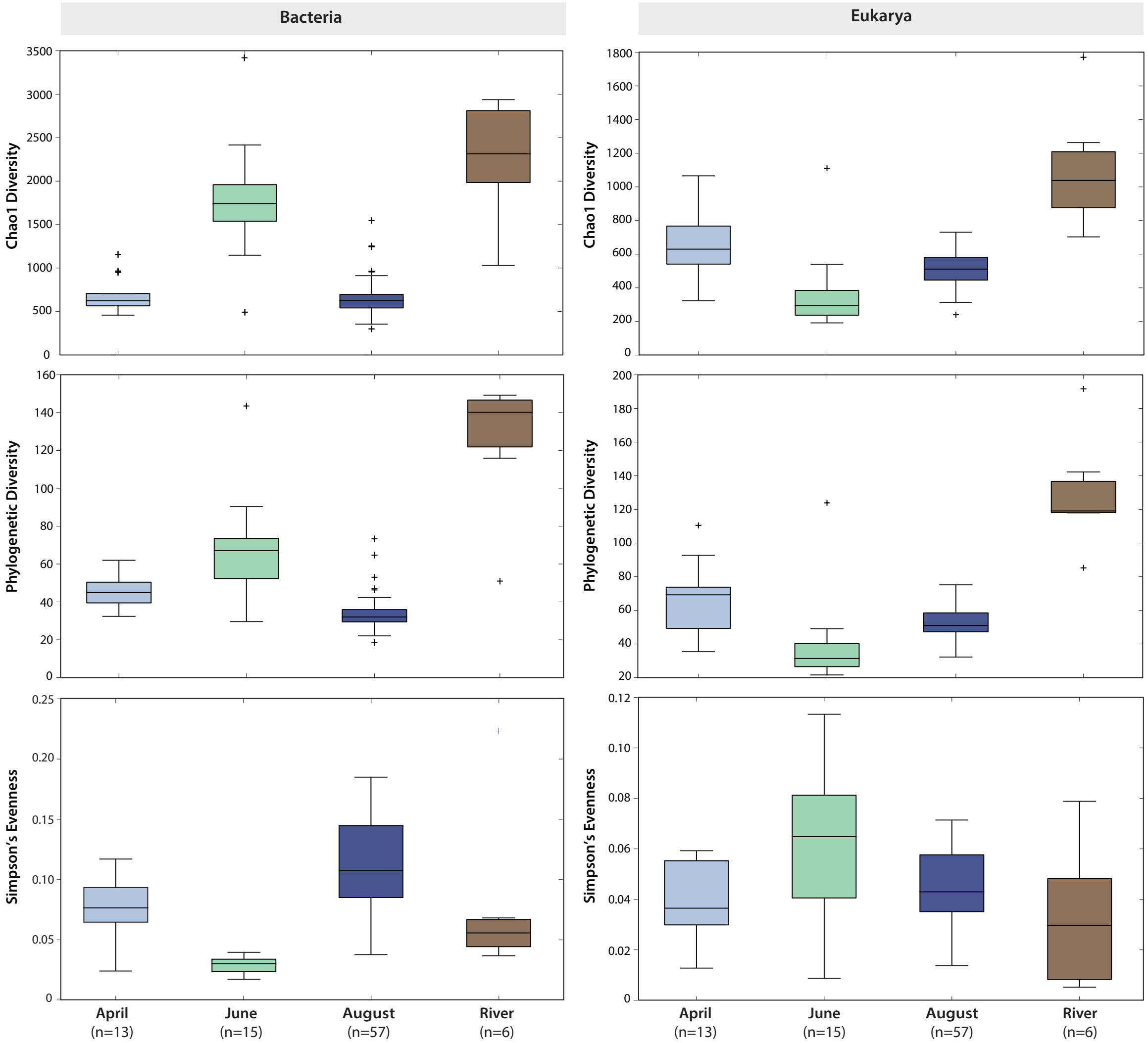

Fig. S1. Boxplots of the distribution of bacterial (left) and microbial eukaryotic (right) alpha diversity among months and the rivers sampled. Three different metrics were used: (top) Chao1 (middle) Phylogenetic Diversity and (bottom) Simpson's evenness. Chao1 and Phylogenetic diversity estimate the number of different taxa in a sample while Simpson's evenness is related to the distribution of reads across the OTUs in a sample.

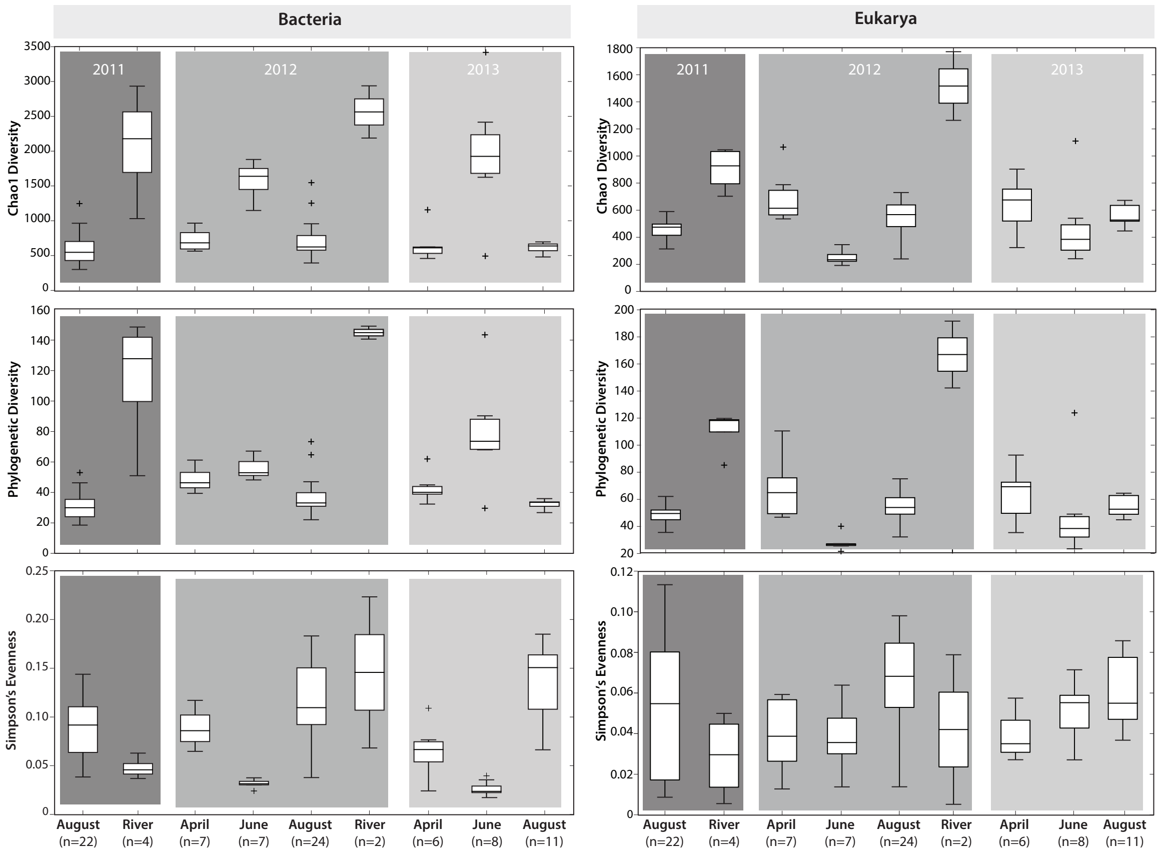

Fig. S2. Boxplots of bacterial (left) and microbial eukaryotic (right) alpha diversity, this time separated by year and month within each year to show interannual variability in diversity among months.

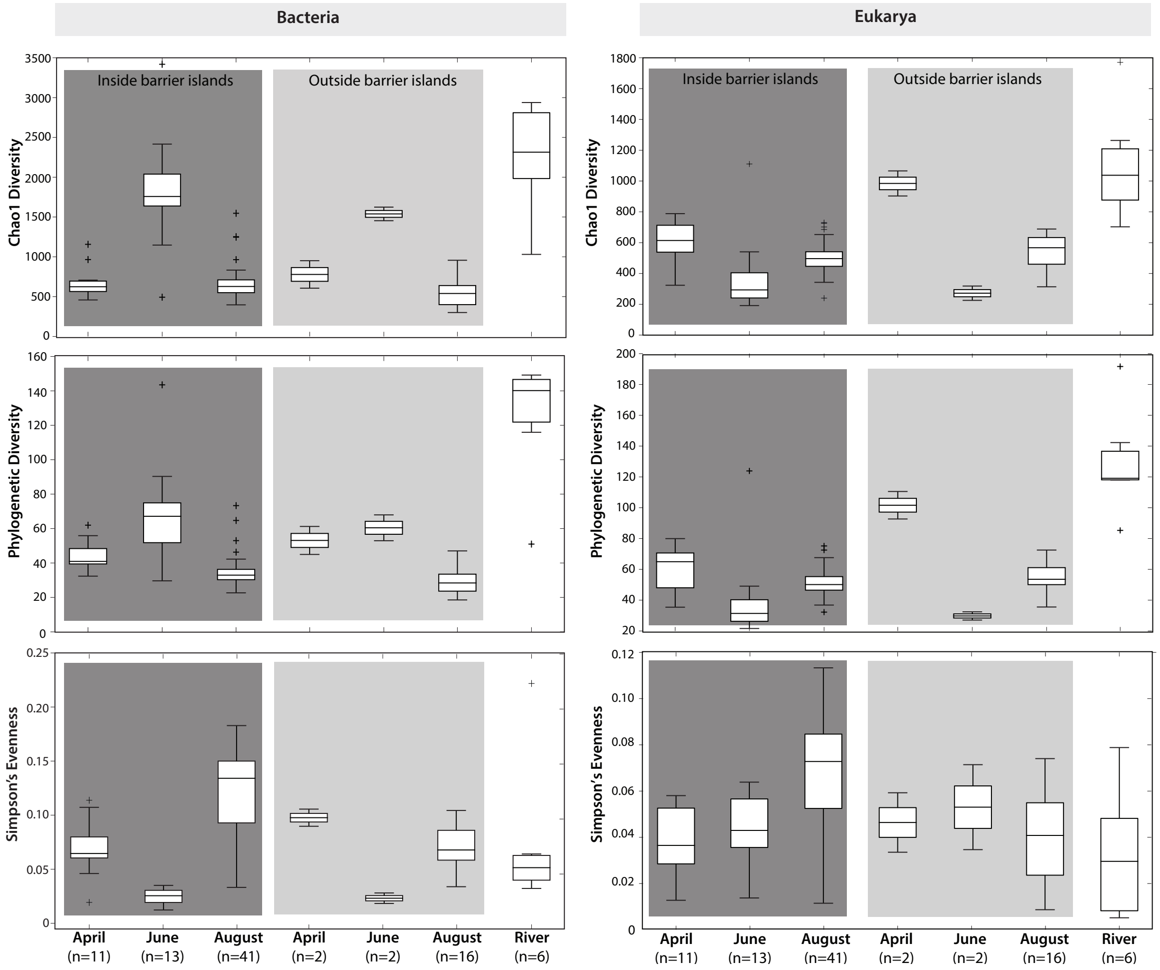

Fig. S3. Boxplots of bacterial (left) and microbial eukaryotic (right) alpha diversity, this time separated by whether the sites were located within or outside the barrier islands. Within this grouping, samples were further grouped by month.

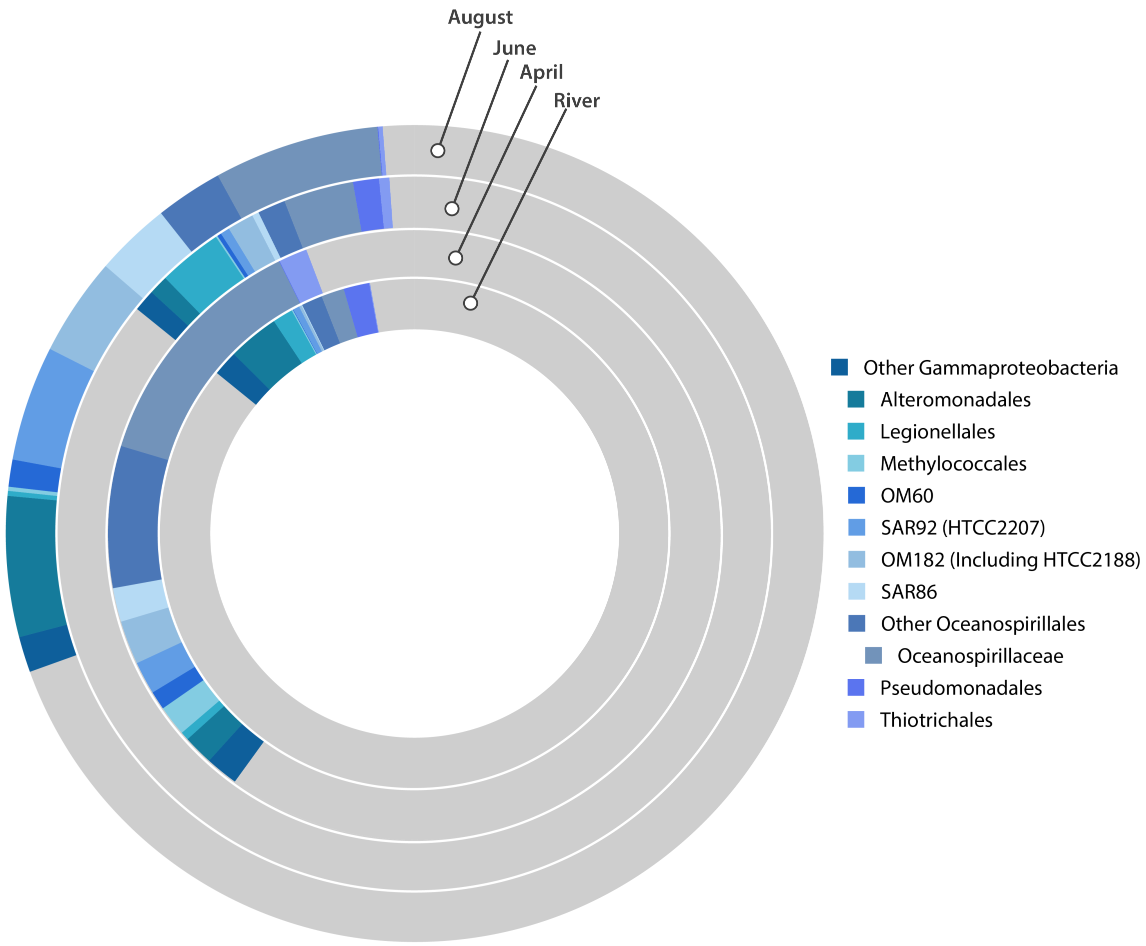

Fig. S4. Taxonomic breakdown of the proteobacterial class Gammaproteobacteria, averaged for each month across all years sampled.

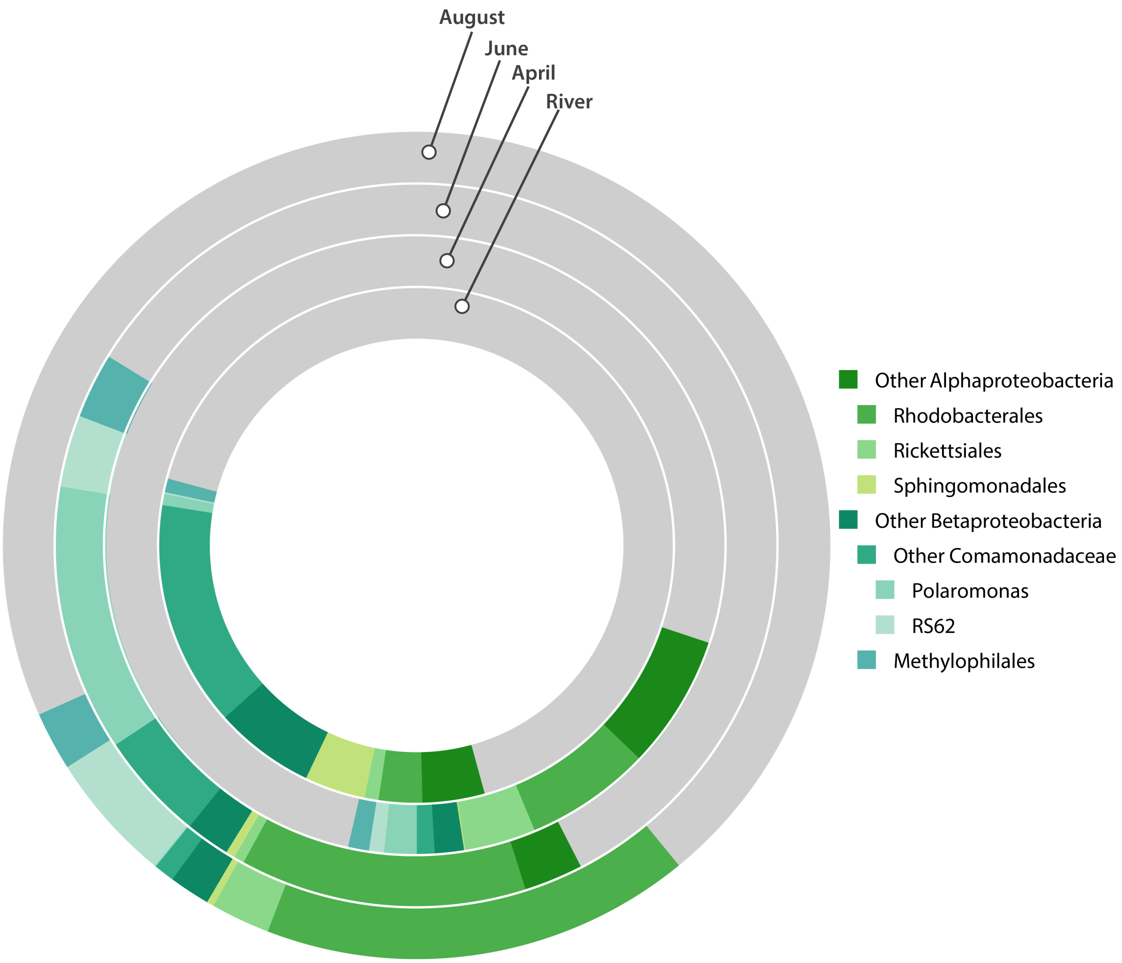

Fig. S5. Taxonomic breakdown of Alphaproteobacteria and Betaproteobacteria classes, averaged for each month across all years sampled.

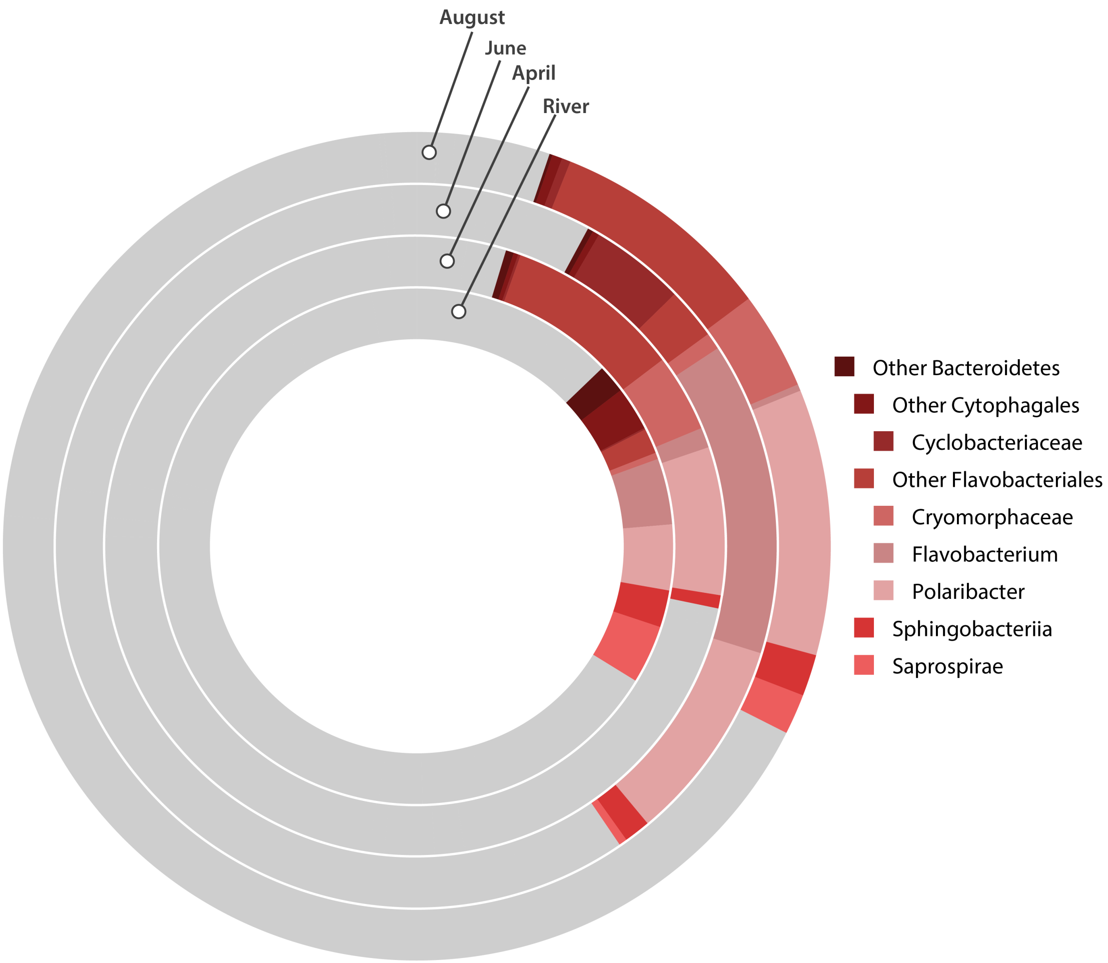

Fig. S6. Taxonomic breakdown of the Bacteroidetes phylum of the Bacteria, averaged for each month across all years sampled.

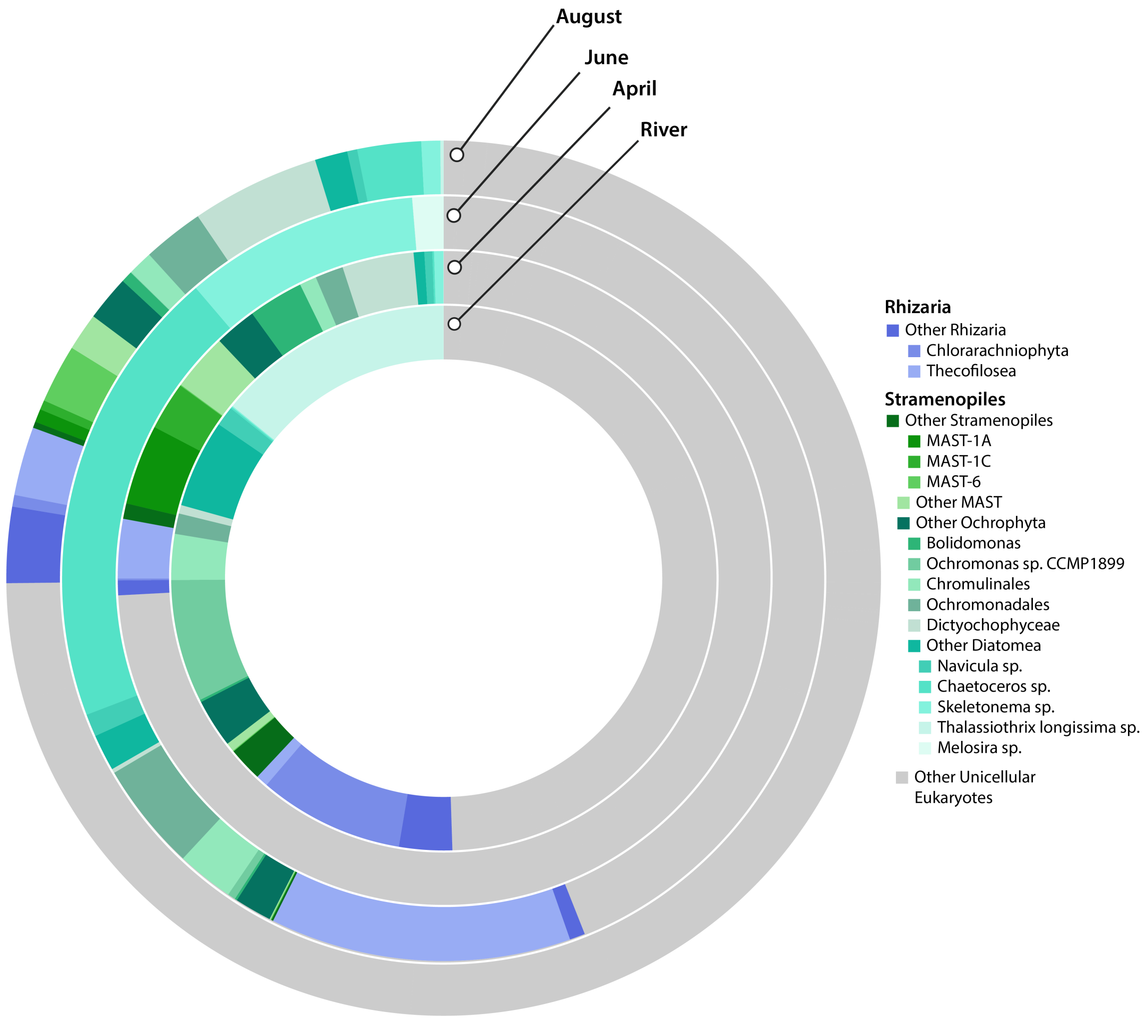

Fig. S7. Taxonomic breakdown of eukaryotic groups Rhizaria and Stramenopiles, averaged for each month across all years sampled.

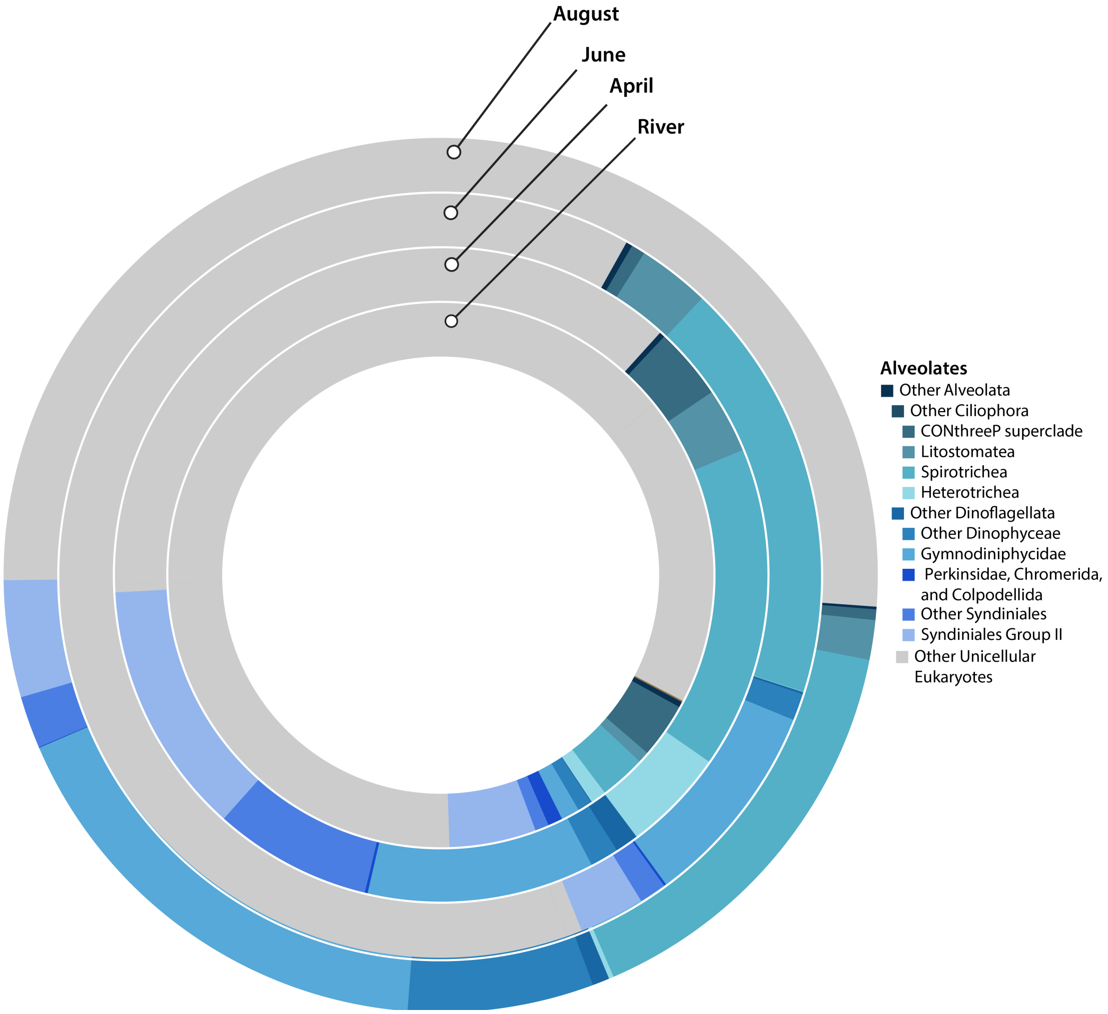

Fig. S8. Taxonomic breakdown of the eukaryotic group Alveolata, averaged for each month across all years sampled.

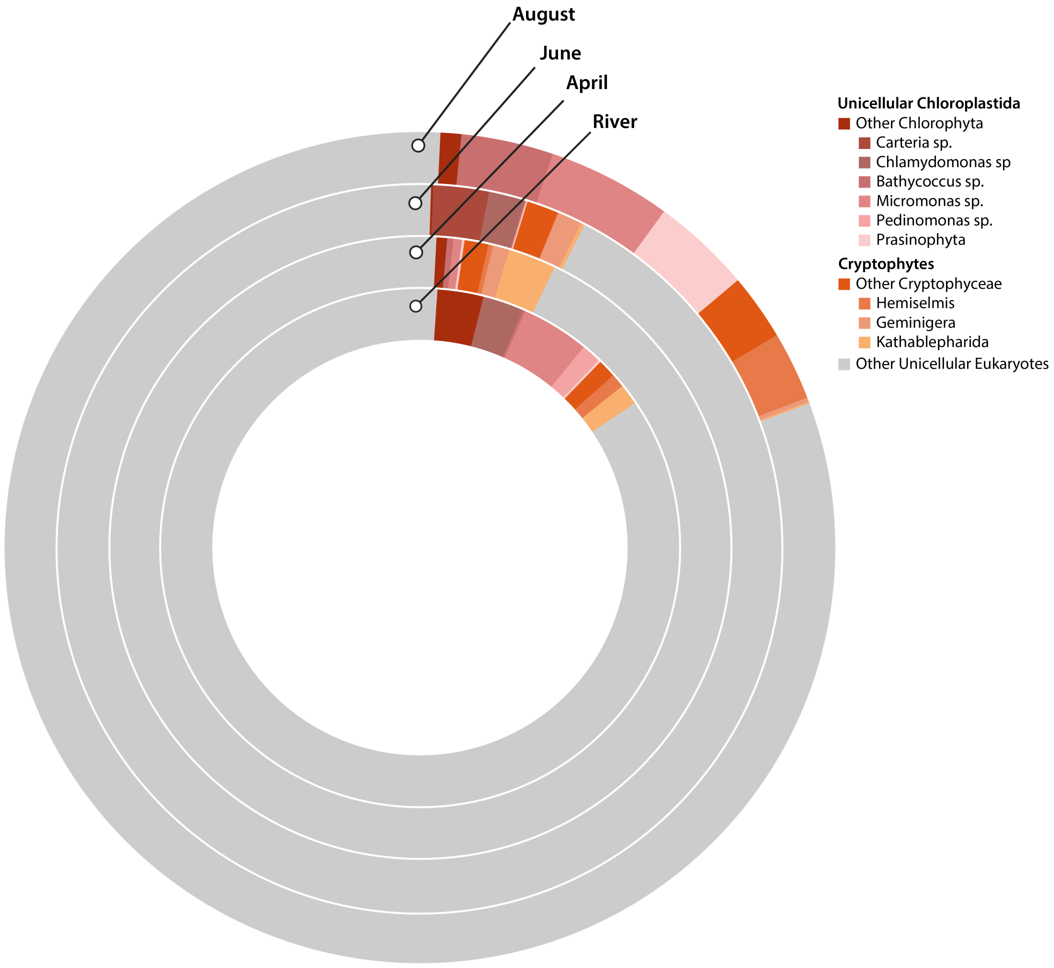

Fig. S9. Taxonomic breakdown of unicellular members of Chloroplastida and Cryptophyta averaged for each month across all years sampled.

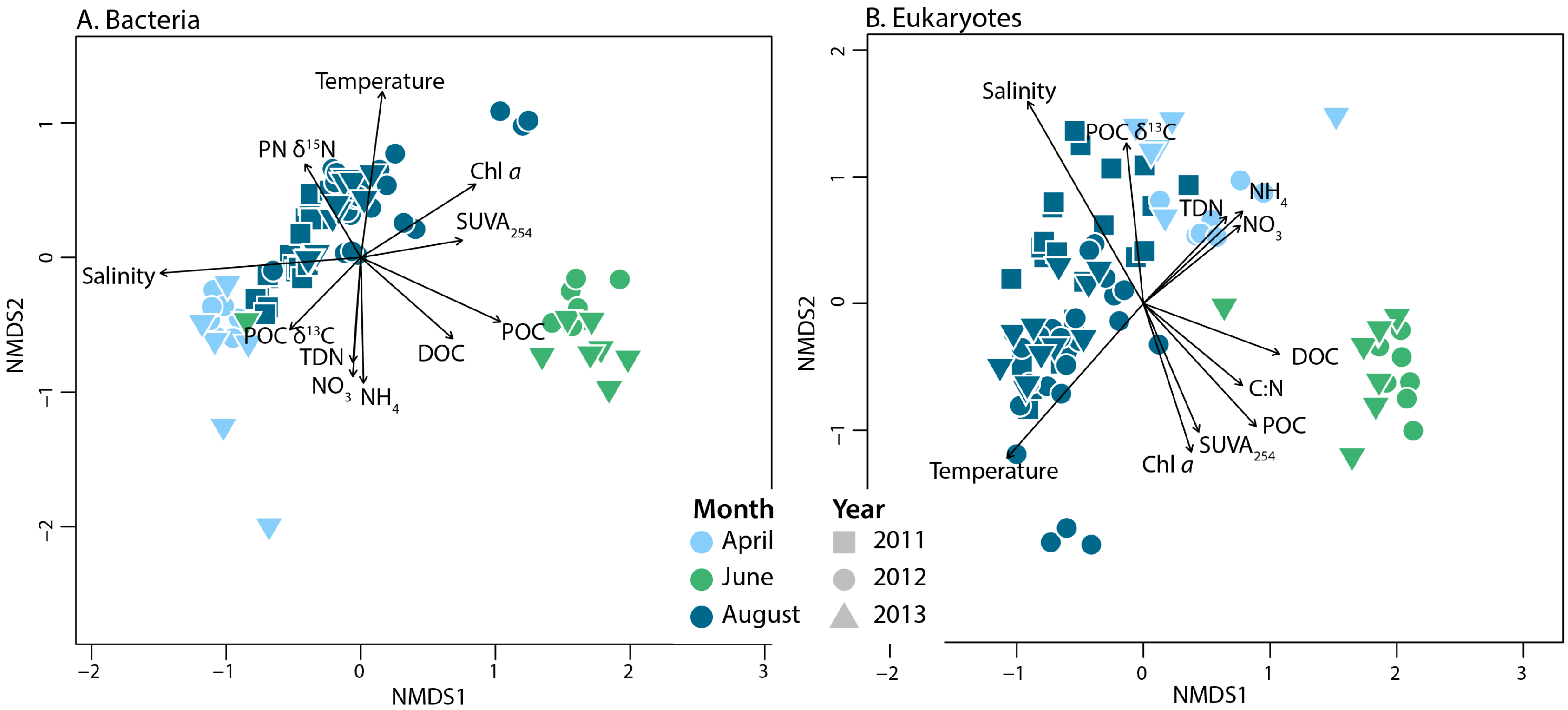

Fig S10. NMDS ordinations of coastal Beaufort Sea (A) bacterial and (B) microbial eukaryotic communities (marine samples only) with vectors representing correlations between physico-chemical variables and each ordination axis. Only vectors for variables with correlations p-values of 0.001 or smaller are plotted.

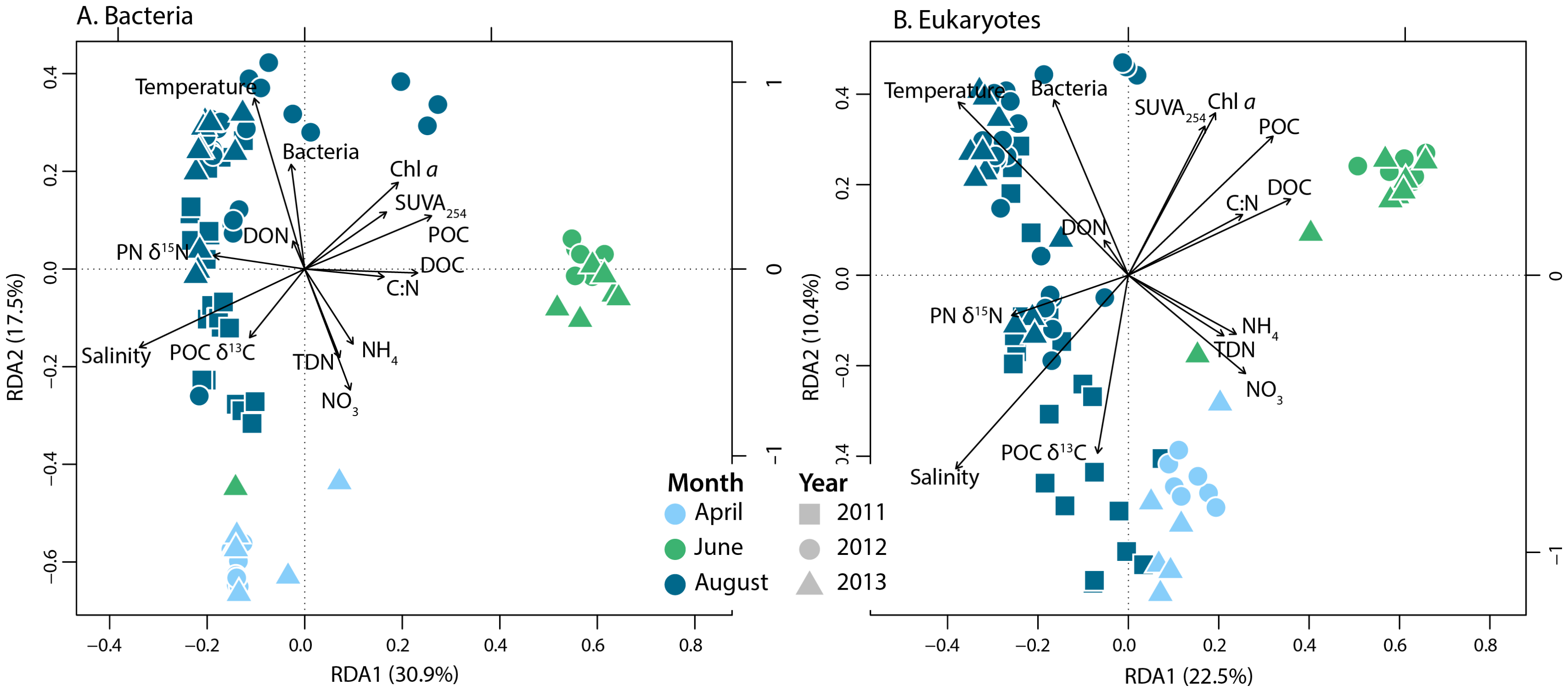

Fig. S11. Redundancy analysis biplots of (A) bacterial and (B) microbial eukaryotic community-environment relationships.

Table S1. Summary of input and output OTUs and environmental variables in monthly co-occurrence networks. For the output OTUs, the percent of the input OTUs is also give in parentheses. The number of significant edges as well as the total number of *possible* combinations of nodes is also given. Finally, we report the percent of these possible combinations that are significant for each network.

	**Input**			**Output**						
**Month**	**Euk**	**Prok**	**Env**	**Euk**	**Prok**	**Env**	**Total Nodes**	**Significant Edges**	**Possible Combinations**	**% Significant Combinations**
**April**	1719	1893	20	628 (36.5)	628 (33.2)	16 (80)	1272	21122	808,356	2.6
**June**	1127	4874	20	196 (17.4)	1010 (20.7)	5 (25)	1211	109143	732,655	14.9
**August**	3183	5046	20	321 (10.1)	331 (6.6)	10 (50)	662	3121	218,791	1.4

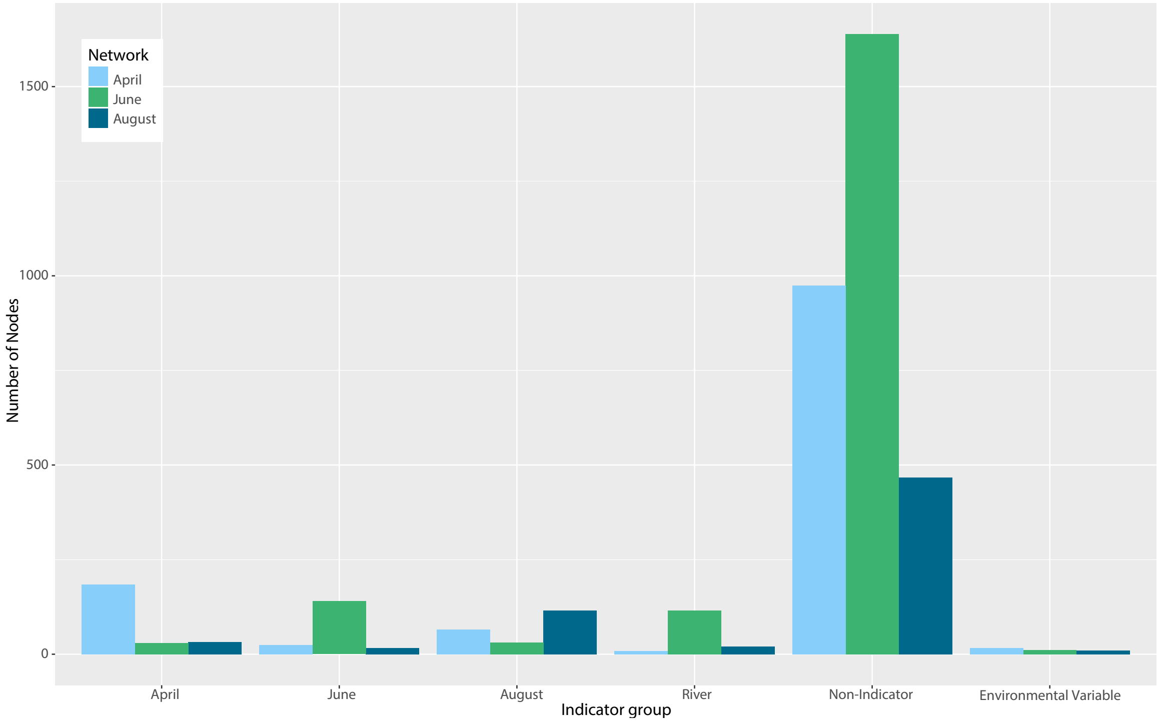

Fig. S12. Distribution of indicator taxa, non-indicator taxa, and environmental variables (x-axis) within each network (colored bars). For example, if you look at the number of April indicator taxa nodes (left-most set of bars), it is clear that the April network contains the most April indicator taxa but that some April indicator taxa are also present into June and August networks.

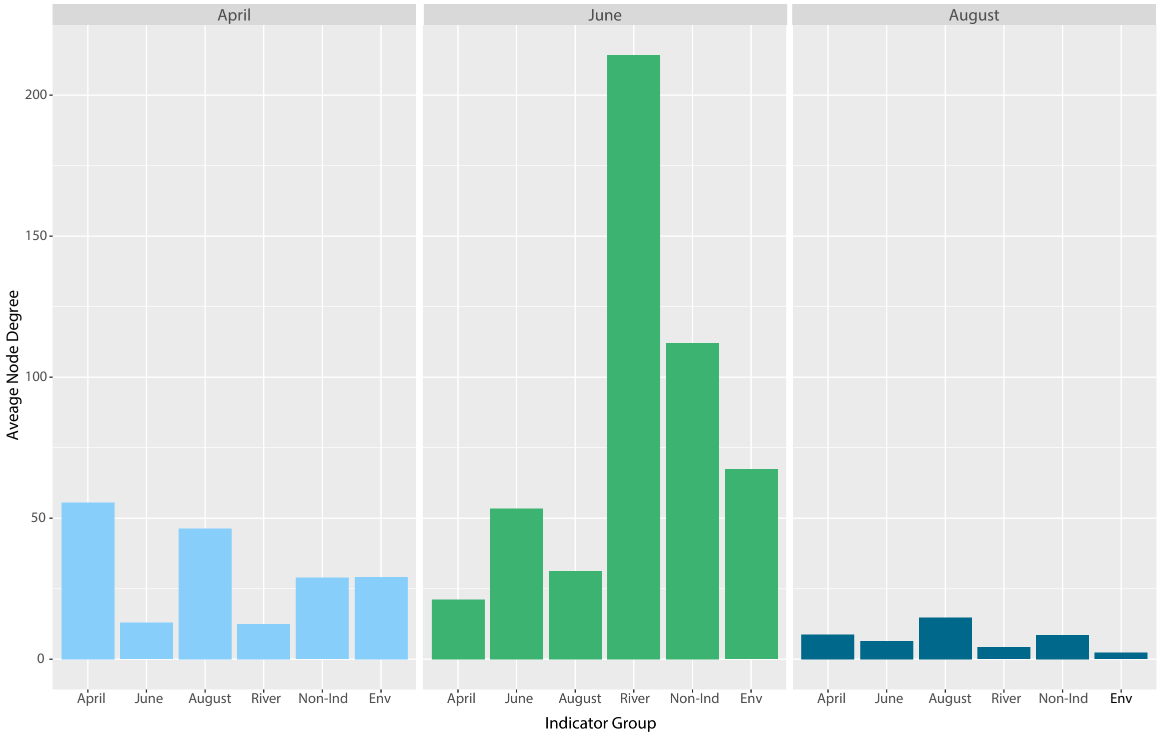

Fig. S13. Average node degree for indicator taxa, non-indicator taxa, and environmental variables within each network. OTUs with higher average node degrees are more connected within a network.

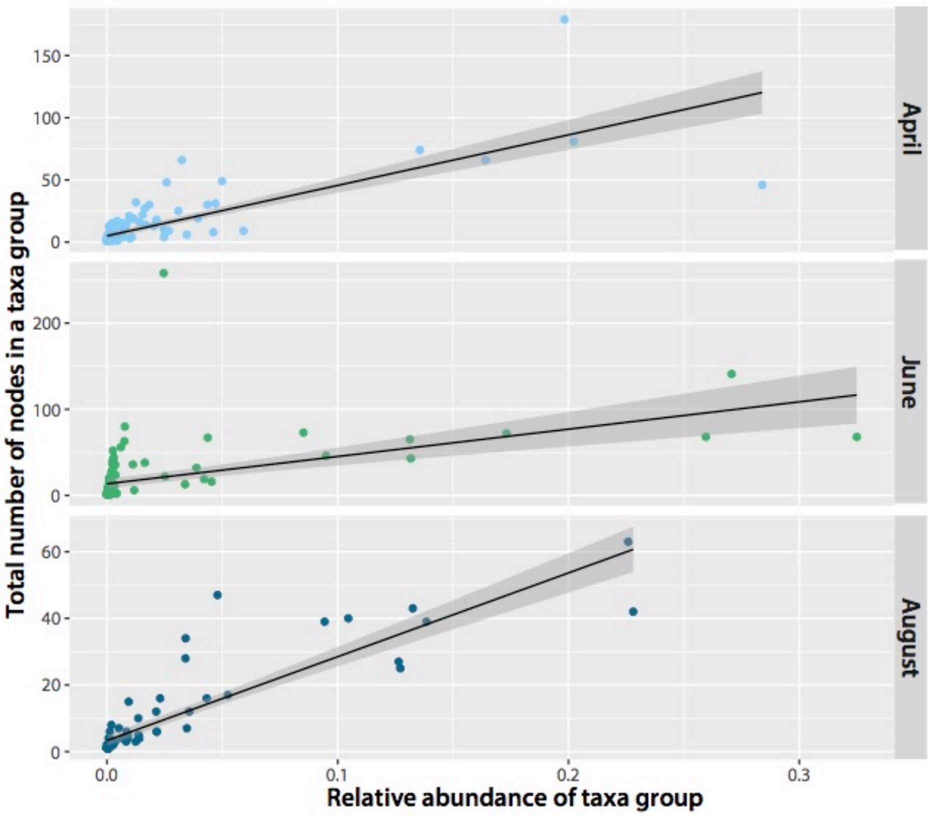

Fig. S14. Relationship between the total relative abundance of a taxonomic group in a month with the number of nodes from that group for each network.

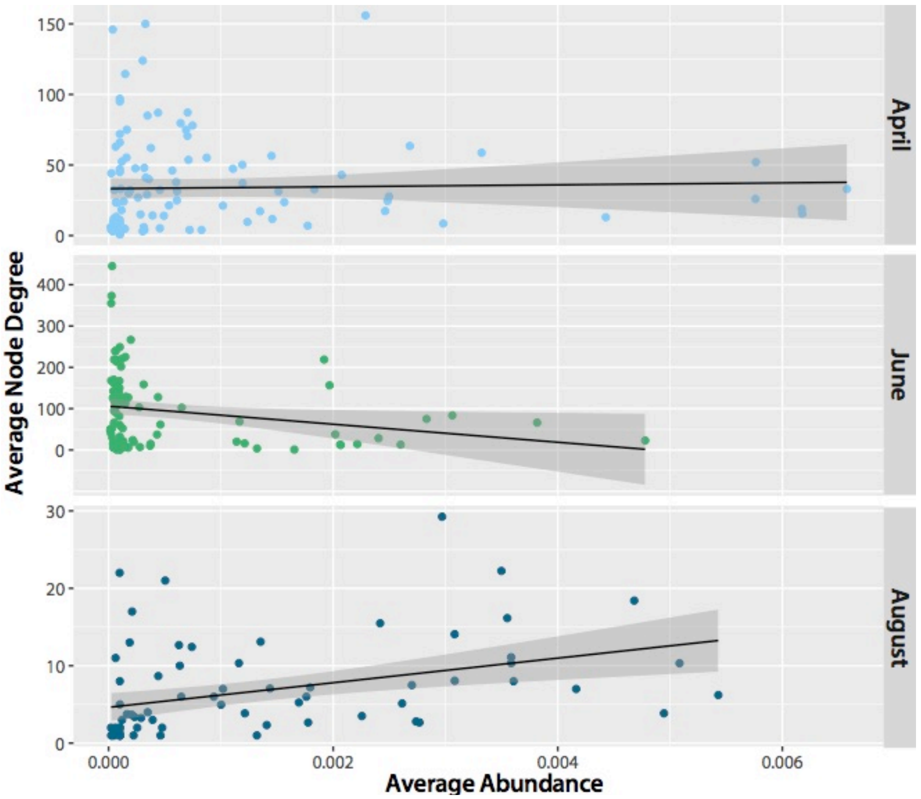

Fig. S15. Relationship between the average abundance of a taxa by month and the average node degree of that taxa within a network.

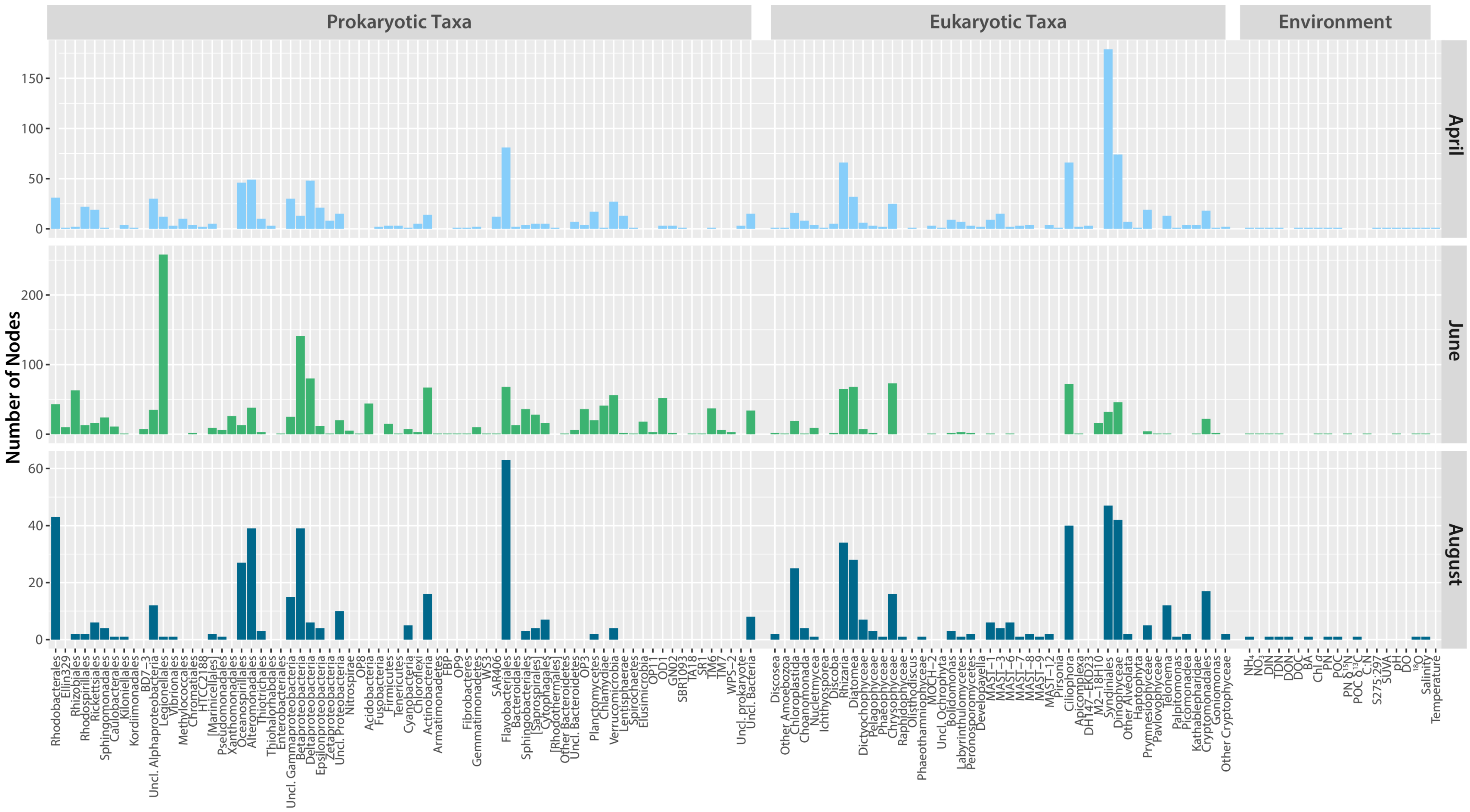

Fig. S16. Node distribution among major taxonomic groups for each network. Environmental variables can only occur a maximum of one time in a network and their inclusion in this figure simply shows whether or not a particular variable had significant relationships within a network. The number of edges associated with each environmental variable is shown in Fig. S19.

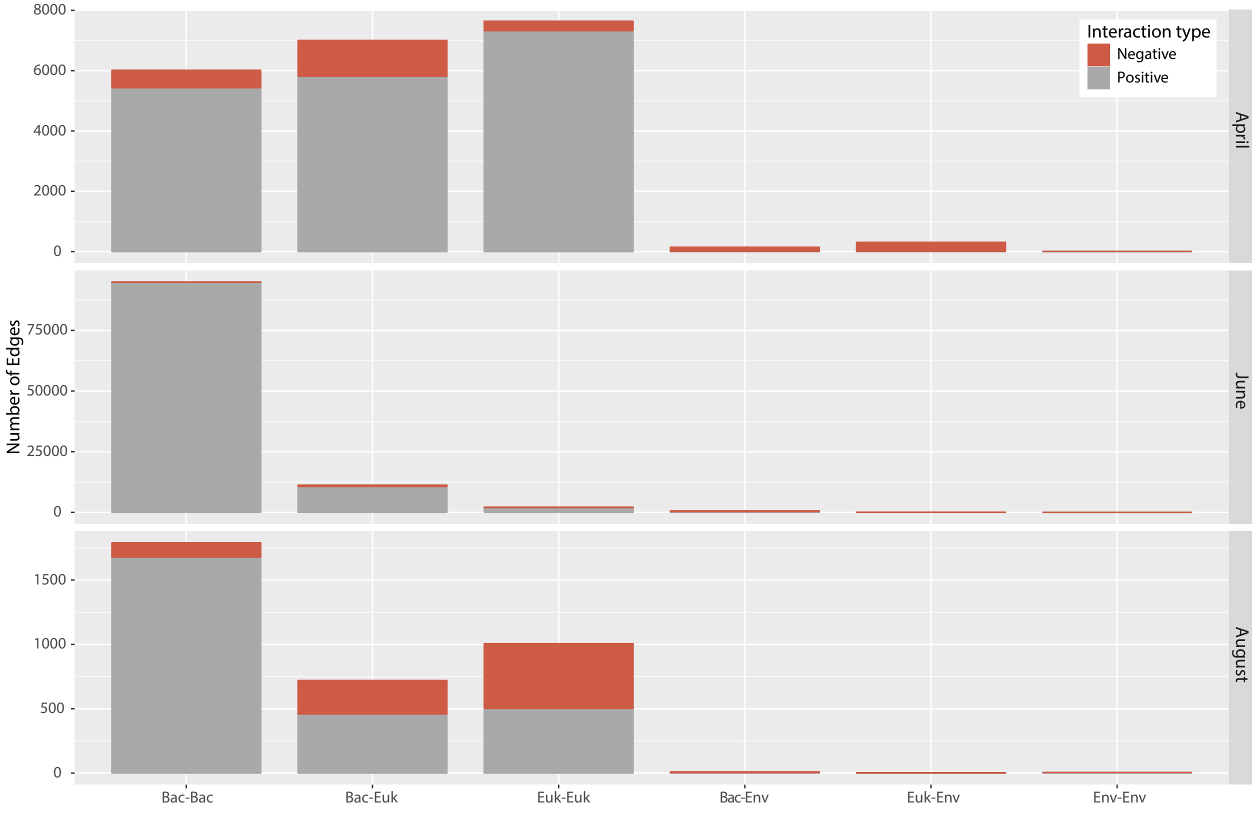

Fig. S17. Number of positive (co-occurrence) and negative (mutual exclusion) edges between bacteria (Bac), microbial eukaryote (Euk), and environment nodes (Env) for each network.

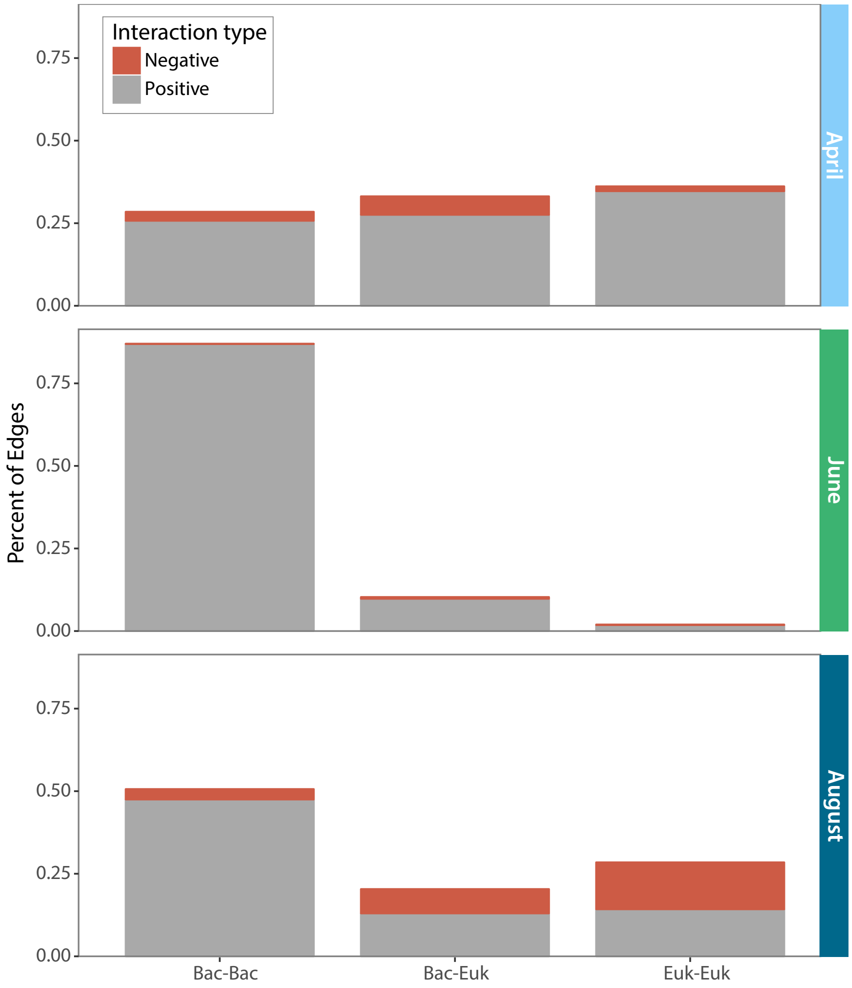

Fig. S18. Distribution of significant relationships between bacteria, bacteria and eukaryotes, and eukaryotes.

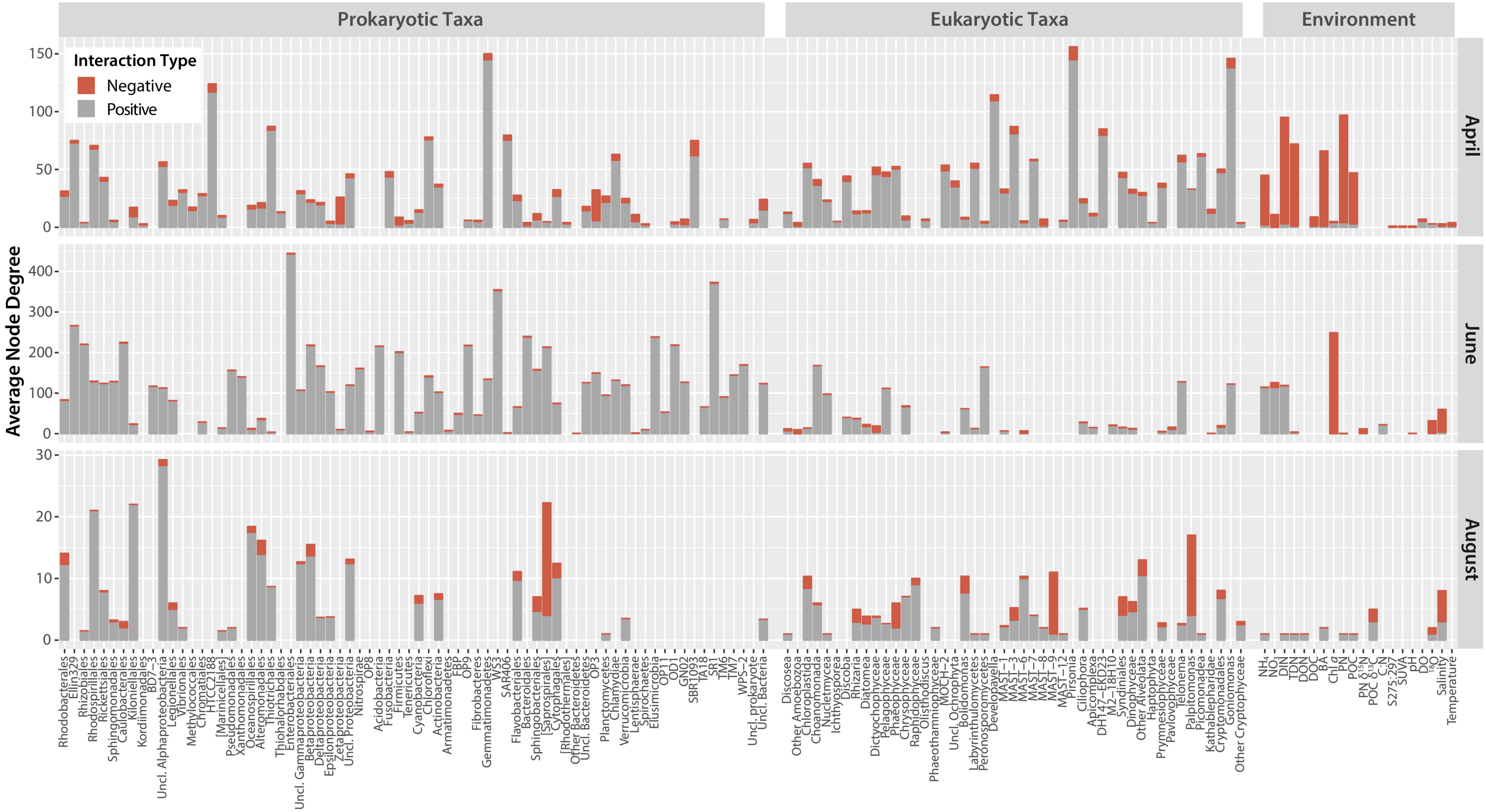

Fig. S19. Average node degree for each major microbial taxonomic group for each network. Bars are further broken down by the average relative amounts of negative (red) and positive (grey) edges for each taxa or environmental variable.
